# Phenotypic and genetic characterization of tomato mutants provides new insights into leaf development and its relationship to agronomic traits

**DOI:** 10.1186/s12870-019-1735-9

**Published:** 2019-04-15

**Authors:** Marybel Jáquez-Gutiérrez, Alejandro Atarés, Benito Pineda, Pilar Angarita, Carlos Ribelles, Begoña García-Sogo, Jorge Sánchez-López, Carmen Capel, Fernando J. Yuste-Lisbona, Rafael Lozano, Vicente Moreno

**Affiliations:** 1 Instituto de Biología Molecular y Celular de Plantas (IBMCP), Universitat Politècnica de València – Consejo Superior de Investigaciones Científicas, Ingeniero Fausto Elio s/n, 46022 Valencia, Spain; 2 Centro de Investigación en Biotecnología Agroalimentaria (BITAL), Universidad de Almería, 04120 Almería, Spain; 3 Facultad Ciencias de la Salud, Universidad Cooperativa de Colombia, Carrera 35#36-99, Barrio Barzal, Villavicencio, Colombia; 4 Facultad de Agronomía, Universidad Autónoma de Sinaloa, Km 17.5 Carretera Culiacán-El Dorado, C.P 80000 Culiacán, Sinaloa Mexico

**Keywords:** Tomato, T-DNA lines, Screening in vitro, Mutants, Leaf development, Organ curvature, Helical growth, Fruit development, Abscission

## Abstract

**Background:**

Tomato mutants altered in leaf morphology are usually identified in the greenhouse, which demands considerable time and space and can only be performed in adequate periods. For a faster but equally reliable scrutiny method we addressed the screening in vitro of 971 T-DNA lines. Leaf development was evaluated in vitro in seedlings and shoot-derived axenic plants. New mutants were characterized in the greenhouse to establish the relationship between in vitro and in vivo leaf morphology, and to shed light on possible links between leaf development and agronomic traits, a promising field in which much remains to be discovered.

**Results:**

Following the screening in vitro of tomato T-DNA lines, putative mutants altered in leaf morphology were evaluated in the greenhouse. The comparison of results in both conditions indicated a general phenotypic correspondence, showing that in vitro culture is a reliable system for finding mutants altered in leaf development. Apart from providing homogeneous conditions, the main advantage of screening in vitro lies in the enormous time and space saving. Studies on the association between phenotype and *nptII* gene expression showed co-segregation in two lines (*P* > 99%). The use of an enhancer trap also allowed identifying gain-of-function mutants through reporter expression analysis. These studies suggested that genes altered in three other mutants were T-DNA tagged. New mutants putatively altered in brassinosteroid synthesis or perception, mutations determining multiple pleiotropic effects, lines affected in organ curvature, and the first tomato mutant with helical growth were discovered. Results also revealed new possible links between leaf development and agronomic traits, such as axillary branching, flower abscission, fruit development and fruit cracking. Furthermore, we found that the gene tagged in mutant *2635-MM* encodes a Sterol 3-beta-glucosyltransferase. Expression analysis suggested that abnormal leaf development might be due to the lack-off-function of this gene.

**Conclusion:**

In vitro culture is a quick, efficient and reliable tool for identifying tomato mutants altered in leaf morphology. The characterization of new mutants in vivo revealed new links between leaf development and some agronomic traits. Moreover, the possible implication of a gene encoding a Sterol 3-beta-glucosyltransferase in tomato leaf development is reported.

**Electronic supplementary material:**

The online version of this article (10.1186/s12870-019-1735-9) contains supplementary material, which is available to authorized users.

## Background

The identification and characterization of mutants has been a determinant factor to gain new insights into genes and mechanisms involved in leaf morphology and color. Chlorophyll-deficient mutants have been exploited in photosynthesis and plant development research [1], while variegated mutants have been mainly used for advancing in the knowledge of chloroplast biogenesis [2]. Considerable progress has also been made in the study of mechanisms related to leaf morphology [3–6]. In this respect, it is worth noting that changes in leaf size and shape not only determine the appearance of the aerial part, but also influence photosynthetic efficiency by modifying light absorption [7, 8], which in turn can affect the yield in a crop species. While Arabidopsis has been the usual model for studying the development of single leaves [6], the tomato is considered the most suitable model for species with complex leaves [3]. Tomato adult leaves generally have three pairs of deeply lobed, large lateral leaflets distributed along the rachis, which develop in a basipetal sequence, the youngest pair of leaflets arising in the basal region of the leaf. Pairs of smaller, less-lobed intercalary leaflets are also born between the large lateral leaflets [7]. The characterization of a number of mutations that increase or decrease the degree of leaf complexity was instrumental in initiating the genetic dissection of tomato leaf architecture. Part of this work has been accomplished thanks to the availability of the collection of spontaneous mutants held at the Tomato Genetics Resource Center (University of California, Davis). Several mutation libraries of tomato cultivars and lines have been obtained by using EMS, fast neutrons and γ-rays [9]. Interestingly, the screening in the field of mutants generated in the genetic background of the inbred variety M82 revealed that leaf shape was one of the most variable morphological traits [10]. Mutant collections have also been generated in the background of Micro-Tom, a dwarf, rapid-growth variety [11–19]. Although these mutant libraries are essential gene resources, the use of spontaneous and induced mutants requires positional cloning and/or mapping-by-sequencing strategies in order to identify the mutated gene [20, 21]. To facilitate the gene identification process, we opted for an insertional mutagenesis-based approach as in this case the inserted element may act as a tag for gene identification. The vector we used was an enhancer trap, since, if the T-DNA is inserted in the appropriate direction near or within an endogenous gene, the reporter expression pattern can mimic that of the latter [22]. Thus, with this strategy it is not only possible to identify mutants based on their phenotypic changes, but also gain-of-function mutants through reporter expression analysis.

Following the generation of several thousands of tomato T-DNA lines, a wide spectrum and great number of dominant and recessive mutants were detected [23]. The evaluation of 4189 T0 transgenic plants and 1858 T1 progenies was performed under greenhouse conditions as at that time we were mainly interested in yield-related traits. However, when looking for alterations in some vegetative traits the screening in vivo is time-consuming, requires considerable space and can only be performed in adequate periods of the year. In order to accelerate the process of detection of this kind of mutants we carried out the screening in vitro of 971 new T1 progenies. Results indicated that in vitro culture is an efficient technique to identify lethal mutants [24] as well as those affected in leaf color, root growth and SAM development (unpublished results). Here we show that in vitro culture is also a quick, efficient and reliable tool for identifying mutants with changes in different aspects of leaf morphology, traits that are usually detected in greenhouse-grown plants at mid- or long-term. Screening in vitro was not only much more efficient in terms of space and time but also allowed increasing sample size, thus improving the selection of recessive mutants with sub-lethality problems as well as those showing a severe growth delay in greenhouse conditions. In vitro culture also facilitated co-segregation analysis through the study of association between the mutant phenotype and the expression of the *nptII* marker gene in a T-DNA insert. In addition, this strategy allowed the study of root development in suitable culture vessels, which provided valuable data in the characterization of certain mutants.

Changes in leaf architecture may be directly or indirectly related to alteration in other developmental traits. This is a promising field in which there is still much to discover although some interesting results have already been published. For instance, the characterization of some tomato mutants led to the conclusion that meristem maintenance and compound-leaf patterning share common genetic mechanisms [25], which could be due to the intimate relationship of SAM and primary leaf development through the blastozone. By characterizing other tomato mutants it has been proposed that shoot branching and leaf dissection are regulated by homologous gene modules [26]. Further investigation on this issue could be relevant from a practical point of view as growth habit is an important agronomic trait in the tomato. It has also been shown that alteration in the *LYRATE* gene not only determines changes in leaf morphology, but also in flower development [27]. Our results on the characterization of a new allele of this gene (*LYRATE-2*), cloned from the insertional mutant *1381-MM* [23], confirmed the observations of these authors and suggested that *LYRATE* could play additional roles in other aspects of reproductive development (unpublished results).

With the aim of shedding new light on the intricate link between leaf morphology and other developmental traits, we carried out the characterization of some of the mutants detected in vitro. As a result, we found tomato mutants putatively altered in synthesis or perception of brassinosteroids, mutations determining multiple pleiotropic effects in vegetative and reproductive traits, lines affected in organ curvature, combining extreme leaf bending with restricted growth of axillary branches, as well as the first tomato mutant with helical growth. Our results also revealed possible new links between mechanisms determining leaf morphology and some agronomic traits, such as root function, flower abscission, fruit development, cuticle structure and fruit cracking. Moreover, the implication of a gene encoding a Sterol 3-beta-glucosyltransferase in tomato leaf development is reported for the first time.

## Results and discussion

### In vitro culture as a tool for screening tomato mutants altered in leaf morphology

**Fig. 1:**
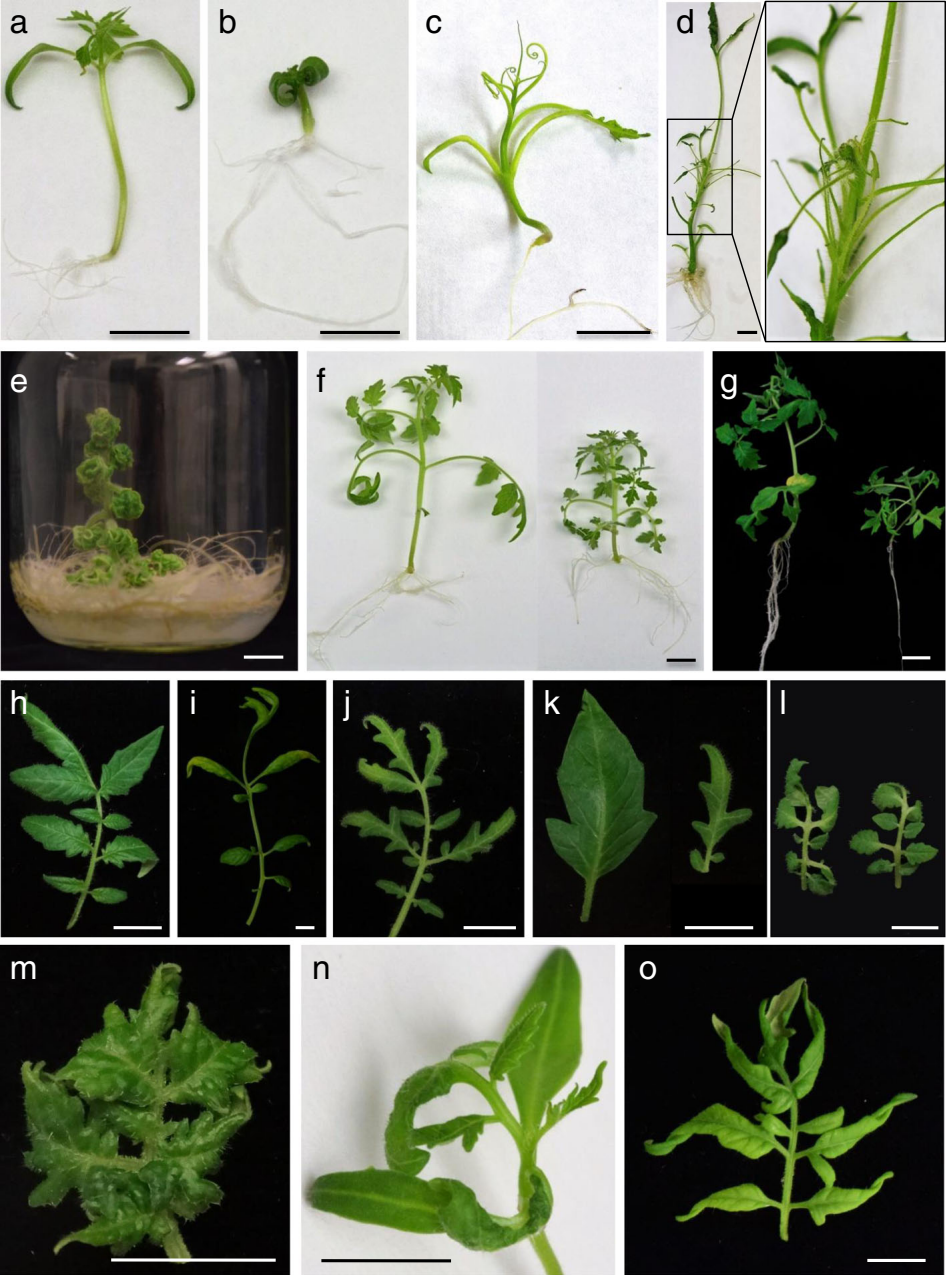
Screening in vitro of mutants altered in leaf development. **a** 12-day-old seedling of tomato cv Moneymaker. **b** Seedling of *2489-MM* (*bst*) mutant showing curly cotyledons, thicker hypocotyls and enhanced root system. **c** Seedling of *1527-MM* (*wl-1527*) showing shoestring leaves. **d** Shoot-derived plants of *150-P73* (*wl-150*) also develop shoestring leaves. **e** Shoot apex-derived plants of the mutant *4196-SP* (*Pwm*) show extremely curly leaves and develop a profuse root system. **f** Shoot-derived plants of Moneymaker (left) and mutant *2733-MM* (right). Note the smaller size of the leaves and the shorter internodes of the mutant. **g** Plants of the mutant *2059-MM* exhibit a smaller leaf size and a less developed root system. **h** Architecture of the fourth leaf of an axenic plant of the tomato cv P73. **i** The mutant *282-P73* (*Art*) was identified in vitro due to the irregular arrangement and reduced width of leaflets. **j** Architecture of the fourth leaf of an axenic plant of the mutant *605-P73*. **k** Leaflet morphology in plants grown in vitro of tomato cv P73 (left) and mutant *605-P73* (right). **l** The leaves of mutant *744-P73* (*Rgb*) are smaller and show a slight or moderate bending. **m** In the mutant *1491-MM* (*Jmr*), leaf wrinkling is particularly intense in the first leaves of shoot apex- and nodal explants-derived plants. **n** The leaf wrinkling in the mutant *2742-MM* (*wao*) is easily observed in the first leaves of seedlings cultivated in vitro. **o** The basal leaves of axenic plants of *2635-MM* mutant show a slight curvature and no lobing. Bar = 1 cm

The screening under greenhouse conditions of a collection of tomato T-DNA-lines allowed us to find a great number of mutants [23]. This kind of long-term evaluation was especially useful to detect mutants altered in reproductive traits (e.g. inflorescence and flower architecture, fruit set, fruit development and maturation), although it represented several years of work as well as considerable greenhouse space. When seeking to discover other types of mutants it would be convenient to implement faster but equally reliable scrutiny methods. Thus, in order to detect tomato mutants with changes in early developmental traits we carried out the in vitro screening of 971 new T1 progenies. This method allowed the rapid detection of several lethal mutants, leading to the identification of a gene (*DXS1*) essential for the survival and development of tomato plants [24]. Results also showed that in vitro screening is a fast and effective method to detect mutants affected in leaf color, root growth and SAM development (unpublished results). We wanted to know if this approach would also serve to achieve early and reliable detection of mutants altered in size, shape or leaf architecture, since these traits are generally evaluated in vivo at mid- or long-term. To reach a conclusion, putative mutants identified in vitro (Fig. 1) were studied under greenhouse conditions. In addition, some mutants previously identified in vivo (Additional file 1: Figure S1) were evaluated in vitro in order to see if certain specific leaf changes (e.g. reduced leaflet lobing, more complex leaves) could also be detected in culture vessels. The comparison of the results in both conditions indicated a high phenotypic correspondence (Table 1), showing that in vitro culture is a reliable system for screening mutants altered in leaf morphology.

Kessler et al. [7] characterized several spontaneous tomato mutants and classified them into four categories: reduced leaflet blade expansion (type I), reduced leaf complexity (II), higher or lower leaflet lobing (III) and increased leaf complexity (IV). In our scrutiny we found new mutants of these four types, as well as some others affected in size, texture and leaf architecture (Fig. 1 and Additional file 1: Figure S1). For instance, tomato mutants with curly or wiry-like leaves were easily detected in seedlings cultured in basal medium (Fig. 1a-e). The use of culture vessels also led to finding mutants with smaller leaves and permitted study as to whether this trait was associated or not to compact growth (Fig. 1f-g). As expected, increased complexity of leaves was much more evident in vivo than in vitro, however mutants with this characteristic can also be detected in culture vessels on the basis of the associated changes in size or morphology of leaflets (Fig. 1h-k). Sometimes, some traits were more extreme in one or another situation. For example, in *744-P73* leaflet bending was moderate in vitro (Fig. 1l) and extreme in vivo. In the case of the mutant *1491-MM* (Fig. 1m), leaf wrinkling was more intense in vitro than in vivo, whereas the opposite occurred with the indentation of leaflet lobes. The ease of detecting in vitro mutants with wrinkled leaves depends on the stage of development at which this trait clearly manifests itself. For example, mutant *2742-MM* was easily detected in vitro since the trait manifests from the first leaf (Fig. 1n). In contrast, for an untrained person, the mutant *2635-MM* could have gone unnoticed in vitro, since the wrinkling is a late characteristic that appears from the fifth-sixth leaf (Fig. 1o). Certainly, changes that only manifest clearly after a certain developmental stage are not easy to appreciate in a culture vessel but, equally, in vivo scrutiny is not free of problems. In our experience, some plants initially selected in the greenhouse as putative mutants had to be discarded after further characterization because their phenotype was due to an environmental change, some occasional problem with the fertigation system or the incidence of a pathogen. Apart from providing homogeneous conditions, the main advantage of in vitro screening lies in the enormous saving of time and space. Furthermore, the possibility of using larger family size in vitro (more than 50 seedlings per T1 progeny, 48,500 in total) rather than in vivo (usually 16 plants per T1 progeny) allowed the discovery of mutants with sub-lethality that probably would not have been detected in vivo (e.g. *272-P73* and *2733-MM*; Additional file 2: Table S1). It is also worth noting that the use of containers with agar medium made it possible to follow root development, which, together with grafting experiments, allowed conclusions to be drawn on whether a mutation mainly affected the aerial part, the root system or both.

Tomato leaves mostly have trichomes of types I and VI [28], while those of some related wild species, such as *S. pennellii*, *S. habrochaites* [29, 30] and *S. pimpinellifolium* [31, 32] develop glandular trichomes of type IV, which synthesize and exude chemical agents acting as defense products against certain pests like *Tetranichus urticae* [33] and *Bemisia tabaci* [34]. Thus, finding mutants altered in type, number or trichome structure could open up the way to performing the genetic dissection of trichome development as well as of certain pest defense mechanisms. In this regard, it is worth noting that the observation of leaves with a magnifying glass in a laminar flow cabinet allowed detection of mutants with changes in trichome development. For example, most type I trichomes of *744-P73* have abnormal terminal cells, leaves of *1491-MM* have fewer type I trichomes in both abaxial and adaxial sides, while those of *912-P73* have a waxy-looking epidermis and fewer trichomes of types I and VI. By contrast, the line 4728-SP develops a huge number of trichomes in leaves and stem (Additional file 3: Figure S2).

Regarding reproductive traits, three recessive mutants developed seedless fruits, eight gave a variable number of seeds and two showed no alteration in reproductive traits. By contrast, all dominant mutants except one were infertile for different reasons (e.g. abnormal flower development, no fruit setting after a long period of culture, seedless fruits). Some dominant mutations generate important pleiotropic effects, which could be one of the reasons why in our collection of tomato T-DNA lines about two thirds of dominant mutants exhibit partial or total infertility. However, it should be noted that this figure is in part due to the high frequency of infertile dominant mutants in two phenotypic categories, one of which is obvious (mutants with seedless fruits) and another which, in principle, was not (mutants with alterations in leaf development). Notably, the greenhouse assessment has shown that many mutants selected for seedless fruit production also exhibited alterations in leaf development, suggesting possible links between both traits.

### Estimation of the number of T-DNA inserts

In mutants with infertility problems (i.e. most of them dominant), the number of inserts was determined by Southern blot (Table 1). When there was no such problem, a segregation analysis was performed to estimate the number of inserts carrying a functional *nptII* marker gene (Additional file 4: Table S2). As expected, the mean number of T-DNA inserts determined by the first method (2.5) was higher than with the second (1.4), since Southern blot reveals the presence of inserts with a non-functional or truncated marker gene that are not detected in segregation analysis. Our results in tomato are similar to those obtained by Jeon et al. [35] with a comparable sample of rice T-DNA lines (2.2 and 1. 4 inserts estimated by Southern and segregation analysis).

### Co-segregation analysis

With respect to other mutant-based approaches, one of the advantages of insertional mutagenesis is that the T-DNA can tag an endogenous gene, greatly facilitating its identification [36]. However, as our collection of tomato T-DNA lines was generated by *Agrobacterium*-mediated transformation, it cannot be ruled out that the phenotype of some mutants is due to somaclonal variation [37]. For this reason, before deciding the best approach to address the cloning of the altered gene, it is necessary to determine whether or not there is an association between the mutant phenotype and a T-DNA insert.

In mutants with no problems of fertility, co-segregation analysis was performed in vitro by studying the association between the mutant phenotype and the expression of the *nptII* marker gene. In order to reach a significant conclusion in statistical terms (*P* > 95–99%), more than 47 (P > 95%) or 72 (*P* > 99%) T1 plants of recessive mutants and more than 15 (P > 95%) or 23 (P > 99%) T1 plants of the only fertile dominant were analyzed (see Methods). As proof of the validity of this method, we used the line *1381-MM* from which we had previously identified a new allele of the *LYRATE* gene [23]. As expected results showed that co-segregation can be admitted with a probability greater than 99%, providing additional evidence that *LYRATE* is the gene tagged in *1381-MM* (Additional file 5: Table S3).

In another seven lines with a single T-DNA insert, the detection of mutant plants sensitive to kanamycin showed an absence of co-segregation (Additional file 5: Table S3). In five of them (*700-P73*, *1458-MM*, *2059-MM*, *2733-MM* and *2742-MM*), χ^2^ analysis indicated that the observed data did not differ from the expected segregation, suggesting a situation of independent assortment between T-DNA insert and mutant allele. By contrast, in line *272-P73* the observed data did not fit the expected segregation, which could be due to partial linkage or more likely to distortion caused by sub-lethality in homozygous plants (see Additional file 2: Table S1). The existence of co-segregation was also ruled out in lines *1425-MM* and *2489-MM* carrying two T-DNA inserts. In *1425-MM*, the progeny from a T1 mutant plant segregated for expression of marker gene (3 kanamycin resistant: 1 kanamycin sensitive) indicating the absence of co-segregation between the phenotype and a T-DNA with a functional *nptII* gene. In *2489 MM*, the progeny from a T1 WT plant was uniformly kanamycin sensitive but segregated for the phenotype (3 WT: 1 M) indicating that there is no association between phenotype and a T-DNA with a functional *nptII* gene. Interestingly, the analysis of the T1 progeny of the dominant mutant *2635-MM* indicated the existence of co-segregation (*P* > 99%). This result encouraged us to carry out further analysis on several T2 progenies that corroborated the initial conclusion.

In the case of mutants with infertility problems, we used a different approach to assess whether a T-DNA insert could have tagged the mutant allele. The strategy was based on the advantages offered by the use of an enhancer trap. In this case if the T-DNA is inserted in the appropriate direction near or within an endogenous gene, the reporter expression pattern can mimic that of the latter [22]. This offers the possibility of identifying gain-of-function mutants in which there is a correspondence between the phenotypic changes and the reporter expression pattern in certain organs. This approach has two main limitations. First, since the enhancers can act at some distance from the coding sequence, it cannot be ruled out that the reporter is expressed even if the T-DNA has been inserted some kilobases downstream. Secondly, if the T-DNA has been inserted in the opposite direction, there is a risk of discarding a line in which the gene has been tagged. Despite these limitations, as will be mentioned below, the association between phenotypic changes and reporter expression pattern strongly suggests that genes altered in three other dominant mutants are tagged by a T-DNA insert.

### Mutants putatively affected in synthesis or perception of brassinosteroids

Brassinosteroids are endogenous plant hormones essential for the normal regulation of multiple physiological processes including cell elongation and division, vascular differentiation, vegetative and reproductive development, and numerous responses to the environment [38]. The characterization of some Arabidopsis mutants provided preliminary information on the complex route of synthesis of these plant hormones [39, 40]. However, it has been proposed that the tomato could be more useful for this purpose since, despite its larger genome size, it seems to have less genetic redundancy than Arabidopsis [41]. For instance, the *Dwarf* gene, codifying a C-6 oxidase of brassinosteroids, was cloned from the tomato *dwarf* mutant some time ago [42]. However, there is no an equivalent mutant in Arabidopsis, most likely due to genetic redundancy [43]. This suggests that by using the tomato, it may be possible to recover novel mutations in brassinosteroid synthesis and signalling that may be difficult to isolate in other species [41].

**Fig. 2:**
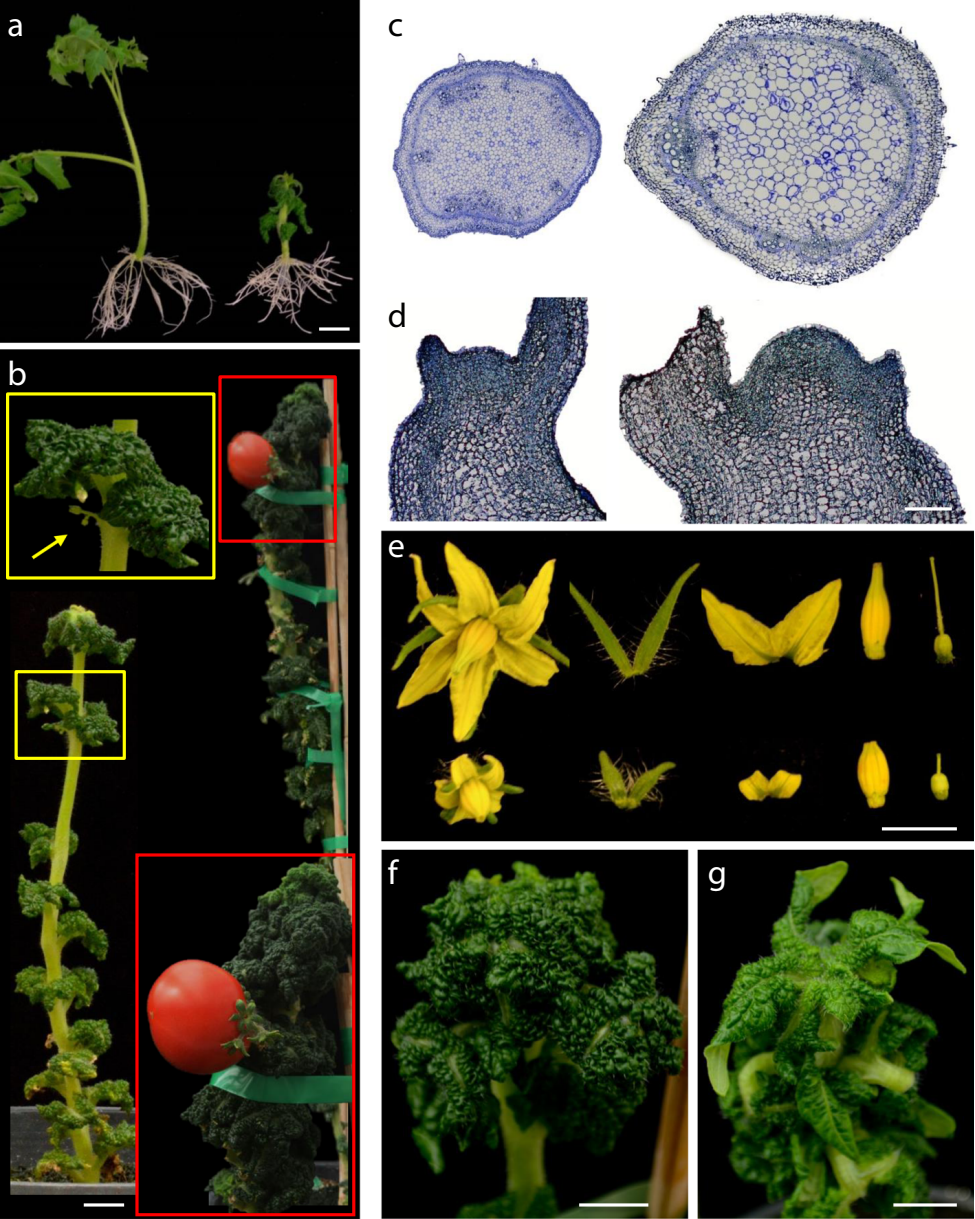
Characterization of the *brassinolide sensitive tomato* (*bst*) mutant. **a** Shoot apex-derived plants of Moneymaker (left) and *bst* mutant (right). Note the extremely wrinkled leaves and the profuse root system in *bst* mutant plants cultivated in vitro. **b** Greenhouse-grown plants of *bst* mutant showed wrinkled dark green leaves and lacked axillary branches. Inflorescences with very few flowers (yellow inset) appeared very late (4 to 5 months) and, despite several trials, only one fruit with few seeds was obtained in a plant cultivated for more than 9 months (red inset). **c** Stem cross sections revealed a larger size in *bst* pith cells, as well as a smaller number and disorganization of vascular bundles. **d** Longitudinal sections showed a larger diameter of the *bst* shoot apical meristem, due to both size and number of cells. **e** The *bst* flowers had shorter petals and sepals, a wider anther cone and a very short style. **f-g** The application of 24-epibrassinolide 1.0 μM every 48 h to mutant plant leaves (**f**) partially rescued the *bst* phenotype (**g**). Bar (a-b and e-g) = 1 cm. Bar (c-d) = 100 μm

Following the screening in vitro a recessive mutant (*2489-MM*) with downward curled thick cotyledons was detected (Fig. 1b). Shoot apex-derived mutant plants showed extremely wrinkled leaves in vitro (Fig. 2a). Despite the lower height and slow growth of the aerial part, the root system developed normally and was even more profuse than that of WT (Figs. 1b; 2a). Greenhouse-grown plants showed wrinkled dark green leaves and lacked axillary branches (Fig. 2b). Stem cross sections revealed a larger size in pith cells, as well as a smaller number and disorganization of vascular bundles (Fig. 2c). Histological sections also showed a larger diameter of the shoot apical meristem, due to both size and number of cells (Fig. 2d). Inflorescences with very few flowers (yellow inset in Fig. 2b) appeared at 4 to 5 months. The flowers had shorter petals and sepals, a wider anther cone and a very short style (Fig. 2e). Despite several trials, only one fruit with few seeds was obtained in a plant cultivated for more than 9 months (Fig. 2b). Overall the phenotype of *2489-MM* resembled that of a *dumpy* (*dpy*) tomato mutant. It has been shown that the application of brassinolide, a precursor of brassinosteroid biosynthesis, rescues the *dpy* phenotype [44]. Therefore, we sprayed 24-epibrassinolide 1.0 μM every 48 h to leaves of *2489-MM*, and observed that the phenotype can be partially rescued (Fig. 2f-g). For this reason, we named this mutant *brassinolide sensitive tomato* (*bst*). As reported in *dpy*, when brassinolide is withheld a reversion to the mutant phenotype is quickly observed in *bst*. To the best of our knowledge, the *DUMPY* gene has yet not been cloned. At present, we are addressing the identification of the *BST* gene by positional cloning and mapping-by-sequencing methods.

**Fig. 3:**
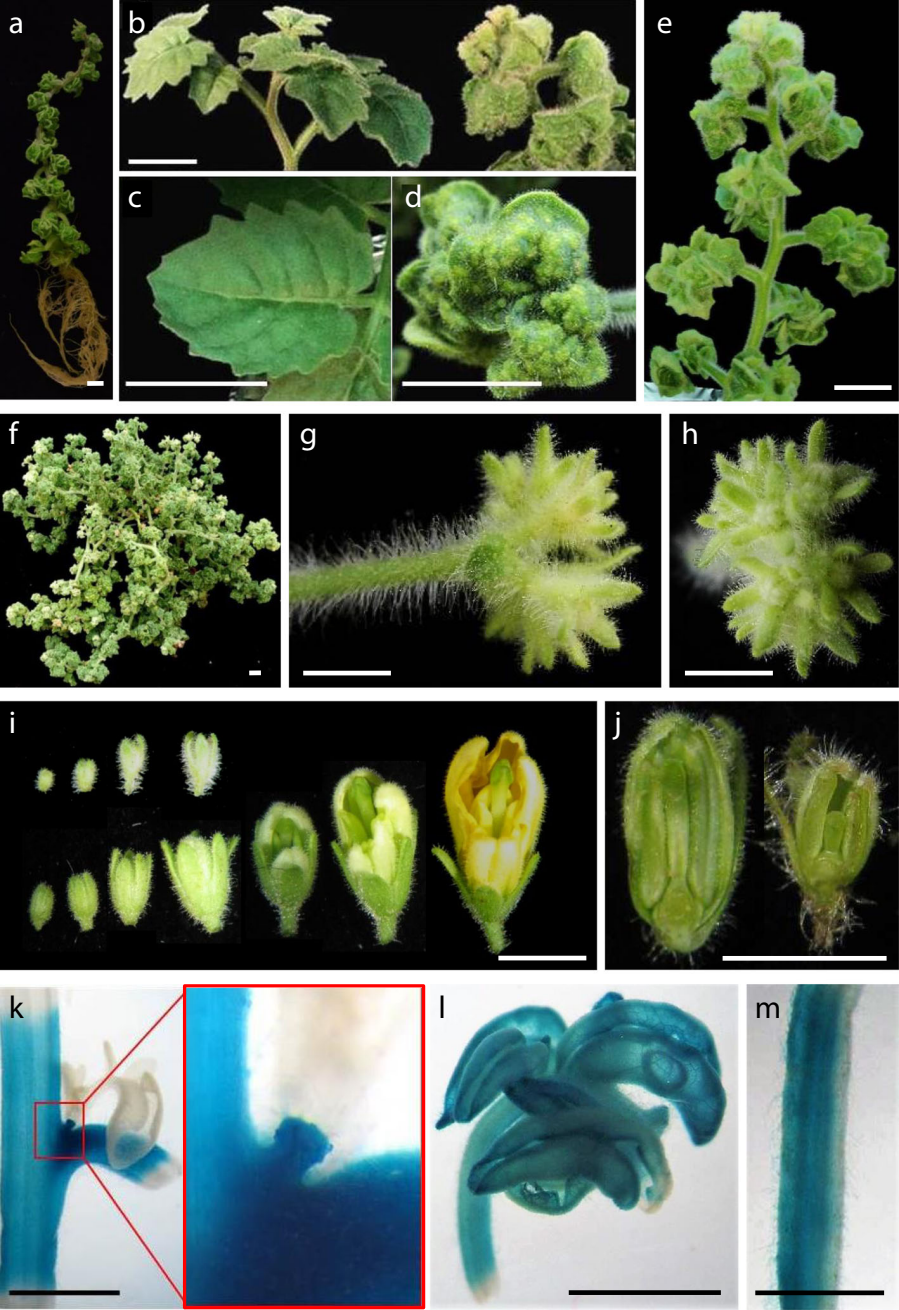
Characterization of the *Pennellii wonderful monster* (*Pwm*) mutant. **a** *Pwm* plants cultivated in vitro show extremely curly leaves and develop a very profuse root system. **b** First leaves of WT (left) and *Pwm* (right) plants. **c-d** Fifth leaf of WT (**c**) and *Pwm* (**d**) plants. **e** Greenhouse-grown plants of *Pwm* grow very slowly and show extremely curly leaves. **f** *Pwm* mutant plant cultivated for 9 months in the greenhouse showing a creeping appearance. **g-h** The *Pwm* inflorescence has a compact appearance since flowers develop side by side in the peduncle terminal part. Note the huge number of trichomes in the peduncle. **i** Unlike what happens in the WT (down), flower development in *Pwm* is interrupted in the floral bud state (up). **j** Longitudinal section of WT (left) and *Pwm* (right) flowers in the floral bud state. **k-m** Expression analysis of the GUS reporter carried by the enhancer trap. A strong reporter expression is observed in axillary buds from which leaves emerge (**k**) as well as in petiole and blade of young leaves (**l**) and rachis (**m**). Bar = 1 cm

The mutant *4196-SP*, named *Pennellii wonderful monster* (*Pwm*), exhibits some traits reminiscent of previously described tomato mutants altered in brassinosteroids such as *curl-3*. The latter is a spontaneous recessive mutant of *S. pimpinellifolium* [45], while *Pwm* is a dominant mutant identified in the screening in vitro (Figs. 1e; 3a) from a set of *S. pennellii* T-DNA lines [46]. *curl-3* shows extreme dwarfism, dark-green curled leaves, delayed development, and reduced fertility [44]. *Pwm* also shows extremely curly leaves (Fig. 3a-e) and growth retardation, but its growth habit is not dwarf (Fig. 3f). As happens with *bst* mutant, in contrast to the slow growth of the aerial part, *Pwm* develops a profuse root system (Figs. 1e, 3a). The inflorescence has an extremely compact appearance since flowers develop side by side in the peduncle terminal part (Fig. 3g-h). Both vegetative and reproductive organs of *Pwm* develop an exacerbated amount of trichomes (Fig. 3b-d and g-h). The mutant is completely sterile since flower development is interrupted in the floral bud state (Fig. 3i-j). At 6 months, the plant has a creeping appearance (Fig. 3f), similar to that of *curl-3* (see Fig. 2f in reference [44]). As with *curl-3*, and unlike what happens in *bst*, treatment with brassinolide was not able to rescue the *Pwm* phenotype (data not shown). The *Curl-3* gene encodes a Leucine-Rich Repeat Receptor Kinase (RLK) which participates in brassinosteroid perception [41]. The total infertility of *Pwm* prevented an allelism test with *curl-3* to see if it is a new *RLK* mutant allele or is affected in a different gene that could also participate in brassinosteroid perception. Total infertility also prevented a co-segregation test, so we carried out reporter expression analysis to know whether there is an association between the mutant phenotype and the reporter expression pattern. A strong reporter expression was observed in axillary buds from which leaves emerge as well as in surrounding tissues of petiole and stem (Fig. 3k). The youngest leaves showed an intense expression in all their tissues (Fig. 3l), while the adult ones had a low expression level in leaf blade and greater in petiole and rachis (Fig. 3m). Overall, results suggest that one of the T-DNA inserts has tagged the gene whose alteration determines the mutant phenotype.

### New *wiry-like* mutants

Tomato *wiry* mutants are characterized by the development of shoestring leaves that lack leaf blade expansion. Genetic analysis of 42 *wiry* mutants revealed four complementation groups, with several alleles producing a wide phenotypic array [47]. The identification of the four *WIRY* genes (*RDR6*, *SGS3*, *AGO7* and *DCL4*) showed that they are involved in biogenesis of trans-acting short interfering RNAs (ta-siRNAs), a class of small RNAs that regulate multiple processes in plants [47, 48]. The *wiry* syndrome results from a failure to negatively regulate *AUXIN RESPONSE FACTORS* (*ARF3* and/or ARF4), which are differentially misregulated in these mutants [47].

**Fig. 4:**
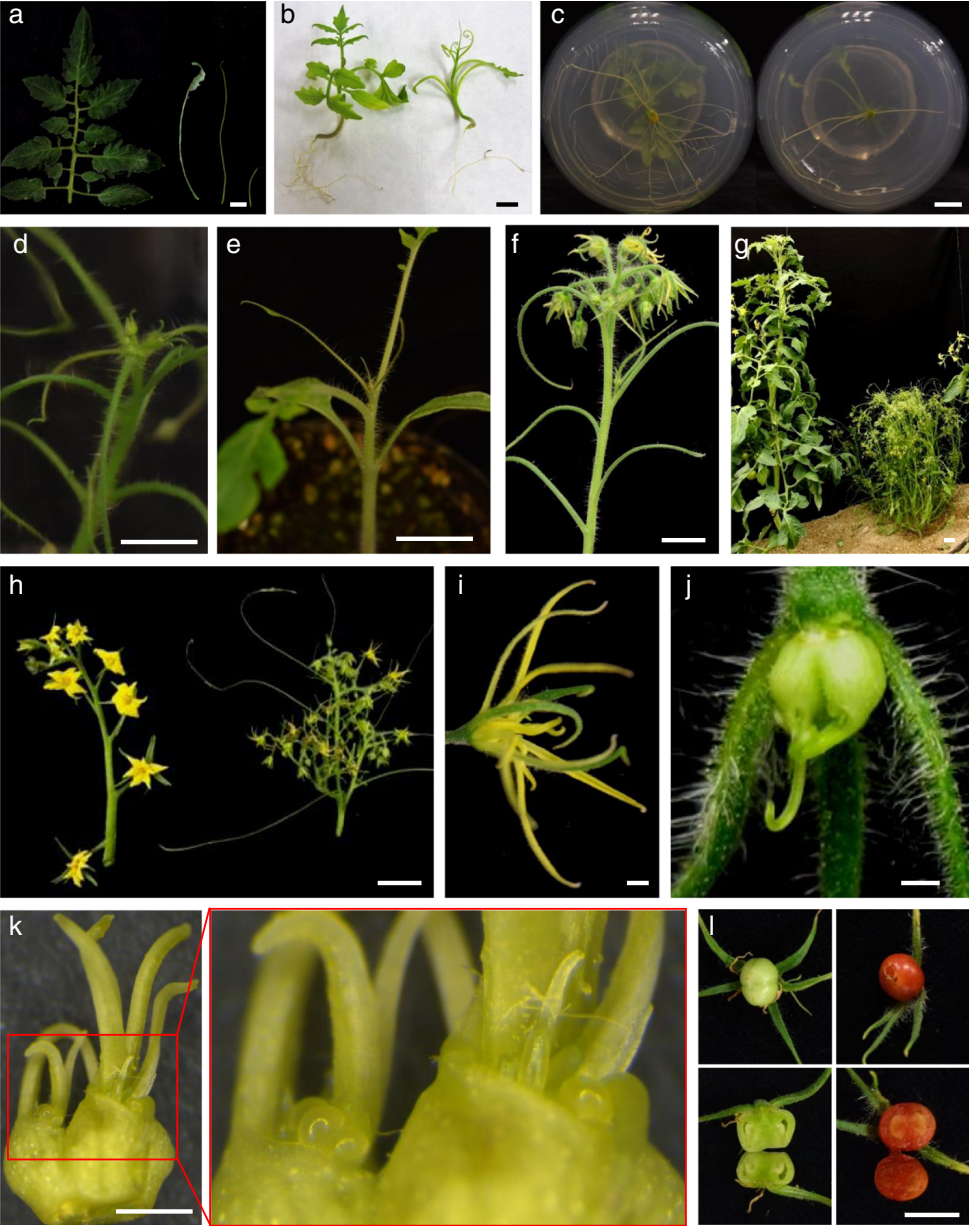
Characterization of the *wiry-like-1527* (*wl-1527*) tomato mutant. **a-b** In mutant plants cultivated both in vivo (**a**) and in vitro (**b**) only the first leaves exhibit a slight expansion of the leaf blade at its terminal end, while subsequent ones display a shoestring shape. **c** Primary root development of *wl-1527* is not affected but the growth of lateral roots is significantly delayed. **d** In plants of *wl-1527* cultivated in vitro early flowering in the shoot apex was observed after just 3 weeks of incubation. **e-f** Early development of a terminal inflorescence is also observed in greenhouse-grown plants. **g** The loss of apical dominance due to early development of a terminal inflorescence promotes axillary branching, giving the adult plants a shrub appearance (right), totally different from that of a wild-type tomato plant (left). **h** *wl-1527* plants develop branched inflorescences, which alternate vegetative and reproductive organs and have a large number of flowers. **i-j** *wl-1527* flowers have thread-like sepals and petals (**i**), a badly fused anther cone (**i**), and a style generally curved downward (**j**). **k** In most cases, the ovary bifurcates and develops vegetative ectopic structures that emerge from the ovules or internal tissues. **l** The fruits of *wl-1527* are small and have placental tissue but lack seminal rudiments. Bar = 1 cm; (i-k) Bar = 1 mm

A new *wiry-like* recessive tomato mutant (named as *wl-1527*) was identified in the in vitro screening (Fig. 1c). In both seedlings and shoot apex-derived plants only first leaves showed a certain degree of blade expansion in the terminal leaflet, while subsequent ones displayed their characteristic shoestring shape (Fig. 4a). The characterization in vitro of *wl-1527* allowed the study of root development, an aspect that to the best of our knowledge has not been described in other *wiry* mutants. Primary root development was not affected but the growth of lateral roots was significantly delayed in both embryo-derived root (Fig. 4b) and adventitious root system (Fig. 4c). Early flowering in the shoot apex was observed after just 3 weeks of incubation in vitro (Fig. 4d). In the greenhouse, early development of terminal inflorescence and subsequent loss of apical dominance (Fig. 4e-f) promoted axillary branching, giving the adult plants a shrub appearance, totally different from that of a tomato plant (Fig. 4g). Apart from their peculiar shoestring leaves, the most distinctive aspect of mutant plants was the development of branched inflorescences, which alternated vegetative and reproductive organs and had a large number of flowers (Fig. 4h). Flowers had thread-like sepals and petals, a badly fused anther cone, and a style generally curved downward (Fig. 4i-j). In most cases, the ovary was bifurcated and developed ectopic vegetative structures that emerged from ovules or internal tissues (Fig. 4k). The fruits, of small size, had placental tissue but lacked seminal rudiments (Fig. 4l).

Mutant *150-P73* (named as *wl-150*) showed a less extreme phenotype as compared to *wl-1527*. Basal leaves had a certain degree of leaf blade expansion, while the following exhibited the characteristic shoestring shape (Additional file 6: Figure S3a). Inflorescences were less branched than those of *wl-1527*, although they also alternated vegetative and reproductive traits (Additional file 6: Figure S3b). Flowers also had thread-like sepals and petals, as well as an open anther cone (Additional file 6: Figure S3c), but no anomalies in the ovary were observed. In fact, unlike most *wiry* mutants, *wl-150* was partially fertile since it produced fruits ranging from small seedless to others of normal size with some seeds (Additional file 6: Figure S3d).

siRNAs act as a defense against some viruses, while other pathogens can suppress siRNA production as part of infection [49]. The phenotypic similarity of sensitive plants attacked by viruses and the wiry syndrome, as well as of the molecular processes involved, can make *wiry* mutants a valuable material for studying plant-pathogen interaction mechanisms [47, 50]. ta-siRNAs have also been involved in regulating developmental processes as well as in stress responses [48]. More recently it has been proposed that *SlAGO7* could represent a useful gene for incorporating into tomato breeding programs, as its overexpression not only determines changes in leaf shape and inflorescence architecture, but also increases fruit yield [51]. Therefore, the identification of the altered genes in these two new *wiry* mutants could be relevant in both basic and applied research.

### Organ curvature

The ability of a plant to keep the growth of its organs in an orderly and established manner is an essential feature not only from an adaptive point of view but also in agronomic terms. The reason why some mutants are not able to maintain the flatness in their leaves or petals has been studied in the *cincinnata* (*cin*) mutant of *Antirrhinum* and similar mutants of Arabidopsis [52–54]. Yet, the mechanisms determining the proper organ growth are still poorly understood in crop species. We discovered a tomato mutant which could bring new insights in this important issue.

**Fig. 5:**
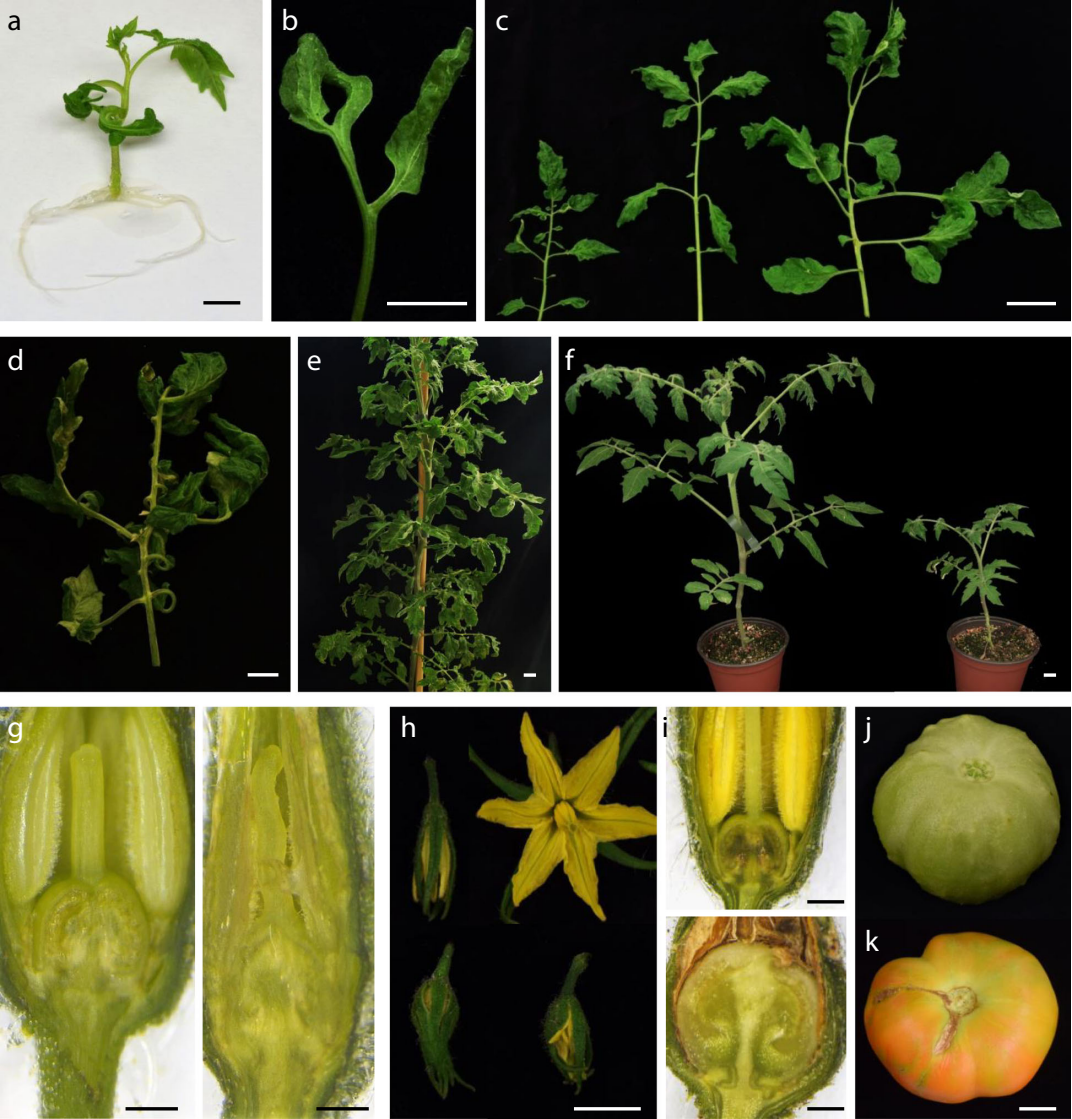
Vegetative and reproductive development of the *wrinkled aerial organs* (*wao*) tomato mutant. **a** *wao* plants cultivated in vitro develop wrinkled leaves and show a moderate curvature of the stem. **b-d** In greenhouse-grown *wao* plants, the degree of leaf blade wrinkling depends on the ontogenetic stage. The first two leaves are saddle-shaped (**b**), the wrinkling decreases in young leaves (**c**), and increases again in the adult ones, although without being as pronounced as in the first leaves (**d**). **e** Adult plant of the *wao* mutant. **f** The WT aerial part grafted on its own root (left) grows much more than when grafted on the mutant root (right) indicating that the gene altered in *wao* also plays a functional role in the root system. **g** The style of the *wao* flower is curved and the anthers develop abnormally. **h** In *wao* flowers, sepals wrap the internal floral whorls, preventing the expansion of petals in anthesis. **i** The conversion of ovary to fruit in *wao* occurs in pre-anthesis. **j** At the mature green stage the cuticle of *wao* fruits is wrinkled, giving it a rough texture. **k** The alteration in cuticle structure most probably explains the tendency to cracking in mature red fruits. Bar (a-f, h, j and k) = 1 cm; Bar (g and i) = 1 mm

The recessive mutant *2742-MM*, named as *wrinkled aerial organs* (*wao*), was detected in vitro for its wrinkled leaves in seedlings (Fig. 1n) and the moderate curvature of the stem in shoot apex- or nodal explants-derived plants (Fig. 5a). In greenhouse-grown plants, the degree of leaf blade wrinkling depends on the ontogenetic stage. The first two leaves are saddle-shaped (Fig. 5b), the wrinkling decreases in young and intermediate leaves (Fig. 5c), and increases again in the adult ones, although without being as pronounced as in the first leaves (Fig. 5d-e). Reciprocal grafts showed that the WT root is not able to rescue the mutant phenotype (not shown) and that the mutant root reduces the growth of the WT aerial part (Fig. 5f). This indicates that the altered gene not only controls leaf architecture, but also plays a functional role in the root system. In addition, *wao* exhibits several changes in reproductive traits. The style is curved (Fig. 5g) and sepals wrap the internal floral whorls, preventing the expansion of petals in anthesis (Fig. 5h). The conversion of ovary to fruit occurs in pre-anthesis (Fig. 5i) which, together with anther degeneration (Fig. 5g), determines the formation of seedless fruits. Interestingly, the cuticle of fruits at the mature green stage is also wrinkled, giving it a rough texture (Fig. 5j). The alteration in cuticle structure is the most probable explanation for the high frequency of cracking (33.2% ± 12.6%) in mature red fruits (Fig. 5k). Thus, the *WAO* gene fulfills multiple roles, including leaf morphology, root function, several aspects of flower development, as well as fruit set and fruit cuticle structure.

At very early developmental stages, the phenotype of the first leaves of *wao* is similar to that described in the *cin* mutant of *Antirrhinum* [52]. The inability to maintain the flatness of the leaf surface in *cin* has been explained by the different progression of the cell-cycle arrest during leaf expansion. In *Antirrhinum* WT, the cell-cycle arrest moves gradually from the leaf tip to the base in a slightly convex fashion, resulting in an elliptical final leaf shape. In contrast, in *cin* the arrest front is concave and initially progresses more gradually down the leaf. This progression results in greater growth in marginal regions, leading to a leaf with negative curvature [52–54]. It is possible that *wao* mutant is altered in a homologous gene of *CINCINNATA* or in related genes of the TCP group [54]. However, there are significant differences between *cin* and *wao*, which could be related to the fact that *Antirrhinum* develops simple leaves while those of the tomato are complex. In *wao*, the first two leaves are completely folded (Fig. 5b), but the leaf blade wrinkling decreases as the formation of a complex leaf progresses (Fig. 5c) and is only accentuated in adult leaves (Fig. 5d). The characterization of *wao* also suggests new connections between leaf development and that of other organs. For instance, although the root growth in *wao* is similar to that of WT (Fig. 5a), grafting experiments have revealed that the root does not function normally. As far as we know, a connection between leaf and root development has not yet been described, so this mutant could constitute excellent material for gaining new insights into common mechanisms regulating aerial and underground organs. In the same way, future studies with *wao* could shed light on the apparent connection between leaf wrinkling and fruit cuticle texture, another aspect not described, and which may have agronomic relevance, as cuticle structure is usually related to fruit cracking tendency.

### Leaf bending and reduced growth of lateral branches

The dominant mutant *744-P73* was selected in vitro due to its smaller leaf size and slight bending of leaflets (Fig. 1l). In greenhouse-grown plants both traits are exacerbated to the point that leaves are about a quarter the size of WT and leaflets are completely bent in on themselves (Additional file 7: Figure S4a-b). All vegetative organs (especially rachis and stem) show a less intense green color. The lateral branches are much shorter than those of WT (Additional file 7: Figure S4c-d) which, together with the development of bent leaves and smaller internode distance, gives the plant a peculiar aspect (Additional file 7: Figure S4e). Most of the compact mutants are characterized by a lower height with respect to WT (e.g., Fig. 1f-g), so they show a vertical compactness. In contrast, *744-P73* exhibits a horizontal compactness, that is, it reaches a similar height to WT but, due to leaf bending and the development of multiple short lateral branches, it seems as if vegetative structures were compressed around the stem (Additional file 7: Figure S4e). The inflorescences have a similar architecture to WT, but they are smaller and only develop 2–3 flowers per inflorescence. The first flowers are abnormal but from the third or fourth inflorescence small flowers appear, from which small seedless fruits develop.

The extreme leaf bending in *744-P73* seems to be due to a greater growth of the abaxial zone with respect to the adaxial. This suggests that the underlying mechanism affecting leaf curvature is different from that of *cin* and *wao*. Apart from that, the most remarkable aspect is the lower growth of lateral branches, which prompted us to name it ‘*Reduced growth of lateral branches*’ (*Rgb*). It has been described that there are common genes regulating leaf development and lateral branching [26]. Unlike what happens in other mutants (e.g. *Lateral suppressor*; [55]), in *Rgb* the growth of lateral branches is not suppressed but restricted, so it could be an excellent material for gaining new insights on the mechanisms regulating lateral branching. It should be noted that the advances in this direction would have both basic and applied interest, since tomato pruning in greenhouses requires hand-labor and involves a considerable economic cost.

### Helical growth

**Fig. 6:**
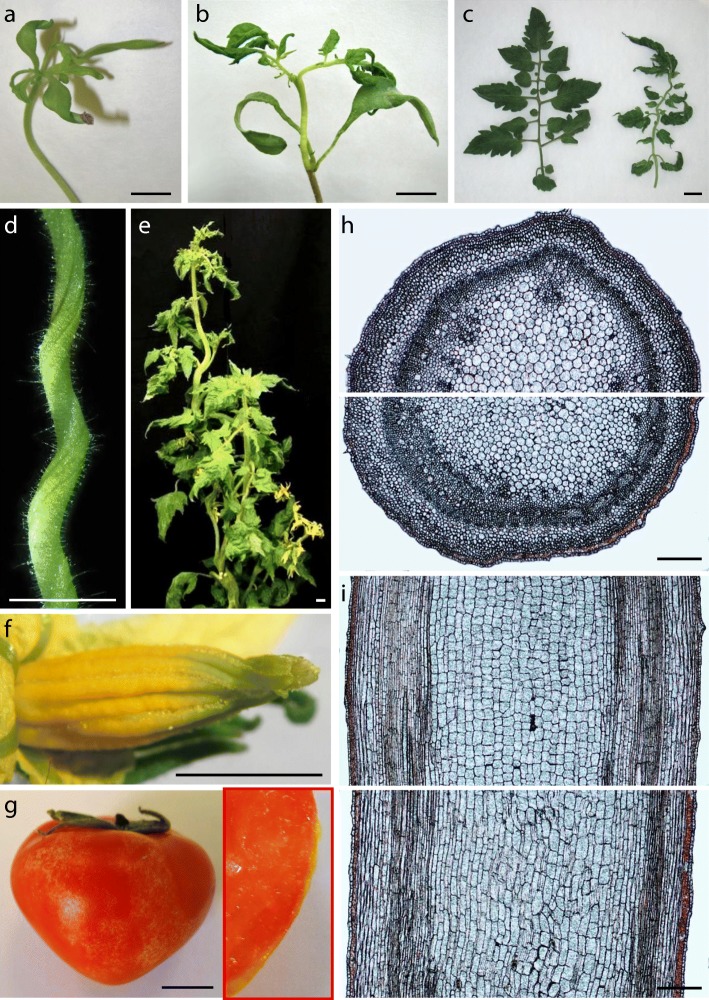
Characterization of *corkscrew* (*crs*), a tomato mutant with helical growth. **a** Slight curvature in the cotyledons and first leaves in *crs* seedlings cultivated in vitro. **b** Helical growth is accentuated in the epicotyl of *crs* young plants cultivated in the greenhouse. **c-d** In *crs* adult leaves the leaflet blade is curved (**c**) and the helical development is still more evident in the rachis (**d**). **e** *crs* adult plant showing helical growth in the stem as well as in the rest of vegetative organs. **f** Helical growth in the three external floral whorls. Note that the anther cone seems normal in its basal part but has a marked helical development at its terminal end. In addition, the sepals and petals curve slightly along their entire surface, except in the terminal part that curves fully towards the abaxial side of the respective floral whorls. **g** The *crs* fruits are of small size and have very few seeds. The fruit skin is variegated (red with yellow spots; see inset on the right) and has a rough cuticle. **h-i** Cross (**h**) and longitudinal (**i**) sections of the wild-type (up) and *crs* stem (down). Cortex cells of the *crs* mutant have a slightly smaller width and similar length. By contrast, mutant pith cells are much narrower than those of WT and twice as long, causing undulations in the cell rows, which most probably explains the helical growth. Bar (**a**-**g**) = 1 cm; Bar (**h**-**i**) = 100 μm

The recessive mutant *1425-MM* is characterized by the helical growth of its organs. Due to the similarity to the pointed metal helix attached to a handle used to extract cork stoppers, we named it *corkscrew* (*crs*). In seedlings cultivated in vitro the trait is manifested by a slight curvature in the cotyledons and first leaves (Fig. 6a). Helical growth is accentuated in the epicotyl of young plants (Fig. 6b). In adult leaves the leaflet blade is curved (Fig. 6c) and the helical development is still more evident in the rachis (Fig. 6d) as well as in the stem of greenhouse-grown *crs* adult plants (Fig. 6e). Helical growth is also manifested in the flower whorls. The sepals and petals curve slightly along their entire surface, except in the terminal part that curves fully towards the abaxial side of the respective organs (Fig. 6f). The anther cone seems almost normal in its basal part but has a marked helical development at its terminal end (Fig. 6f). The fruits, of small size (diameter of 3.29 ± 0.10 cm vs 4.80 ± 0.09 cm in WT; height of 2.61 ± 0.04 cm vs 3.91 ± 0.11 in WT) and with very few seeds, are variegated (red skin with yellow spots) and have a rough cuticle (Fig. 6g).

Two recessive mutations in Arabidopsis, *spiral1* and *spiral2*, reduce anisotropic growth of endodermal and cortical cells in roots and etiolated hypocotyls, and induce right-handed helical growth in epidermal cell files of these organs [56]. It has been proposed that the microtubules are involved in the expression of the helical phenotype although, paradoxically, in the right-hand-growing *spiral* mutants the microtubules are arranged in left-hand helices [57]. Similarly, in *tortifolia1*, which is allelic to *spiral2*, the right-handed helical growth correlates with a complex reorientation of cortical microtubules [58]. In contrast, in the Arabidopsis *tortifolia2* mutant the helical growth does not depend on cell division patterns but involves twisting of isolated cells [59]. The recessive mutation of maize *tangled-1* causes cells to divide in abnormal orientations throughout leaf development. Surprisingly, although mutant leaves grow more slowly in comparison with WT, their overall shape is normal at all stages of growth [60]. Recent studies with this mutant led to a proposal that the normal growth of maize depends on a combination of proper division plane orientation and mitotic progression [61]. To investigate the mechanism determining helical growth in the *crs* tomato mutant, flow cytometry studies were carried out. No differences in the mixoploidy pattern in different parts of the stem of *crs* and WT were found (data not shown), suggesting that mitotic progression is not responsible for the helical phenotype of *crs*. Subsequently, cross and longitudinal sections of the stem were performed to evaluate the cell size in both cortex and pith (Fig. 6h-i). Cortex cells of the *crs* mutant had a slightly smaller width (21.4 ± 0.8 μm as compared to 26.7 ± 1.2 μm in WT) and similar length (139.3 ± 9.2 μm compared to 141.2 ± 10.9 μm in WT). By contrast, mutant pith cells are much narrower than those of WT (41.0 ± 6,3 μm versus 67.5 ± 3.7 μm) and twice as long (162.5 ± 14.3 μm versus 82.5 ± 3.8 μm), causing undulations in the cell rows, which most probably explains the helical phenotype. Therefore, in contrast to the *spiral1* and *spiral2* mutants of Arabidopsis in which the helical growth is associated with a reduced anisotropic growth [56], in the *crs* tomato mutant the phenotype seems to be due to the opposite mechanism, that is, an increase in cellular anisotropy.

### Fruit set

Fruit set rate is a determining factor in the yield of a tomato cultivar. Despite its agronomic importance, knowledge about mechanisms regulating this process is still fragmentary. Similarly, little is known about the existence of possible links between leaf and fruit development, or about the effect that certain modifications of leaf morphology may have on fruit yield due to changes in the source-sink relationship.

The recessive mutant *700 P73* (*necrotic and small leaflets*, *nsl*) was selected in vitro because of the smaller size of leaves, as well as for a hypersensitive response determining necrotic spots in leaf blade. (Additional file 8: Figure S5 a-b). In greenhouse-grown plants adult leaves have an enormous number of small leaflets that also exhibit necrotic spots in the leaf blade (Additional file 8: Figure S5 a-b). Despite these profound alterations in leaf development, the reproductive development is relatively normal. In particular, there are no changes in flowering time or flower architecture (not shown), or in the number of flowers per inflorescence, fruit morphology or seed formation. However, a decrease in quantitative parameters related to the number of fruits per inflorescence, fruit set rate and fruit size is observed (Additional file 8: Figure S5 d). Results suggest that the lower yield of mutant plants is not due to a direct effect of the gene on flower and fruit development, but more likely to an alteration in source-sink relationship due to the modification in leaf development.

**Fig. 7:**
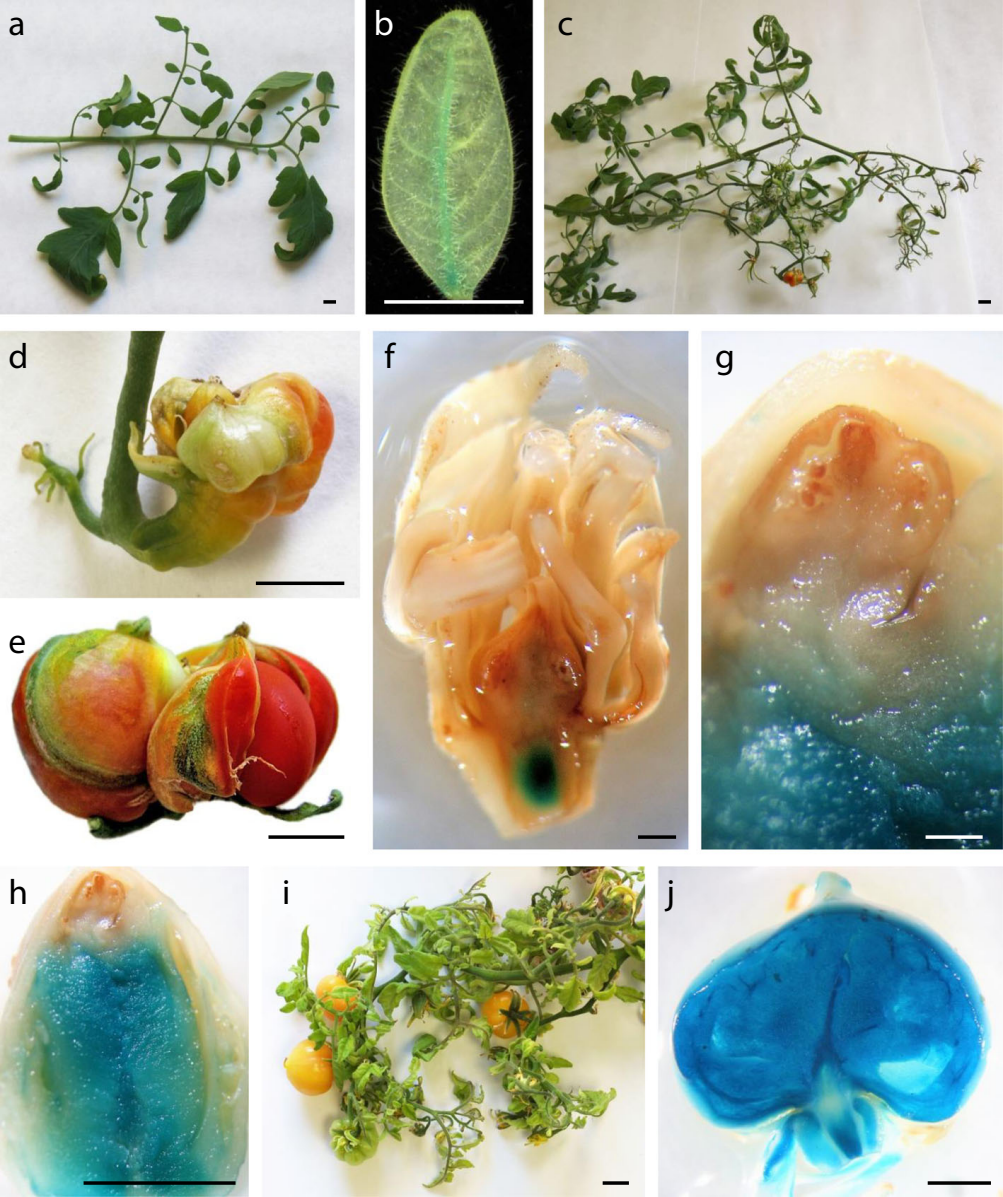
Characteristics of the tomato mutant *Altered in all reproductive traits* (*Art*). **a** *Art* leaves show an irregular arrangement and size of leaflets. **b** Expression of the GUS reporter carried by the enhancer trap in the leaf vascular bundles. **c** The first inflorescences are of enormous size and combine reproductive and vegetative traits. **d-e** Most of the *Art* fruits are totally aberrant. **f-h** The abnormal fruits do not arise from the ovary, but develop in an ectopic way in the union zone of the pedicel and the ovary base. Note the intense GUS reporter expression in the zone where the ectopic development of most of the fruits occurs. **i** When *Art* plants are kept in the greenhouse for more than 8 months, some small and seedless fruits that do not reach maturity develop. **j** In the fruits that develop in old plants, GUS expression is very intense in all tissues. Bar (a-e and i-j) = 1 cm; Bar (f-h) = 1 mm

The dominant mutant *282-P73* was identified in vitro due to the irregular arrangement and reduced width of leaflets (Fig. 1i). The leaf phenotype is still more exacerbated in greenhouse-grown plants (Fig. 7a), but the most remarkable features of the mutant are the multiple alterations in reproductive development, which prompted us to name it *Altered in all reproductive traits* (*Art*). *Art* is an early flowering mutant, which is an agronomically interesting trait (Additional file 9: Figure S6). The first inflorescences are of enormous size and indeterminate, since they combine reproductive and vegetative traits (Fig. 7c). Most of the fruits are totally aberrant (Fig. 7d-e), although if plants are kept in the greenhouse for more than 8 months, some small and seedless fruits that do not reach maturity are developed (Fig. 7i). When analyzing the development of aberrant fruits, we observed that, in contrast to what might be expected, they do not arise from the ovary, but develop in an ectopic way in the union zone of the pedicel and the ovary base (Fig. 7f-h). In previous work, we characterized a tomato semi-dominant mutant (*Arlequin*, *Alq*) whose most relevant feature is that the sepals become fruits. As a matter of fact, in *Alq* homozygous plants sepal-derived fruits acquire all the characteristics of a sink and mature like normal fruits, i.e. those which are ovary-derived [62]. We also demonstrated that *Alq* mutation promotes a gain-of-function phenotype caused by the ectopic expression of *TAGL1* gene [63]. Most likely in *Art* something similar determines the mutant phenotype. The expression of the GUS reporter carried by the enhancer trap is not only located in the leaf vascular bundles (Fig. 7b) but is particularly intense in the union of the pedicel and the ovary base, which is where the ectopic development of most of the fruit occurs (Fig. 7f-h). Similarly, in the fruits that develop with a degree of normality in old plants, GUS expression is very intense in all tissues (Fig. 7j). Overall, the results suggest that *Art* is another gain-of-function mutant in which the only T-DNA insert has tagged a gene that plays a role in leaf morphology, inflorescence development and fruit setting.

### Alteration in abscission zones

**Fig. 8:**
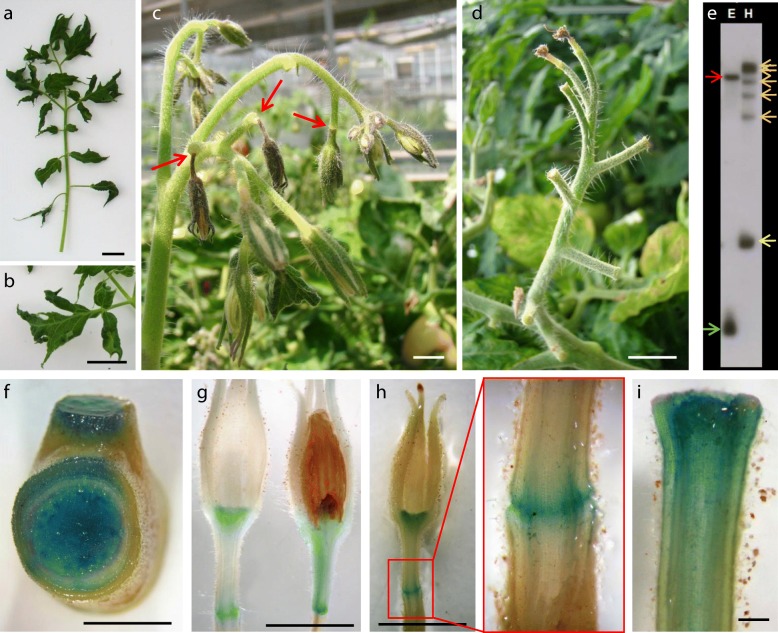
*Jointmore* (*Jmr*), a tomato mutant with an early and generalized activation of floral abscission. **a-b** Leaves of *Jmr* plants cultivated in the greenhouse are almost serrated (**a**) and show deeply indented margins (**b**). **c-d** Early activation of floral abscission. The pedicel abscission zone is activated in flower bud or pre-anthesis stages (**c**), promoting flower detachment from the inflorescence (**d**). **e** Southern blot analysis of *Jmr* genomic DNA digested by restriction enzymes EcoRI (**e**) and HindIII (**h**) and hybridized with the NPTII-FA probe. Red arrow: FA-EcoR1; Green arrow: NPTII-EcoRI; Orange arrows: NPTII-HindIII; Yellow arrow: FA-HindIII. **f** A strong GUS reporter expression is observed in leaf rachis as well as in the stem pith. **g-h** GUS expression is particularly intense in the pedicel abscission zone as well as in the zone where the abscission zones of the flower internal whorls lie. Note in **g** and **h** the absence of the three flower internal whorls due to activation of the corresponding AZs. **i** GUS expression is maintained in the pedicel abscission zone after flower detachment. Bar (**a**-**h**) = 1 cm; Bar (**i**) = 1 mm

Abscission is the process whereby plants shed some of their organs at abscission zones (AZs) either during normal development or in response to tissue damage and environmental stress. In the tomato, the flower and fruit AZ is located in the middle of the flower pedicel. Two recessive mutations, *jointless* (*j*) and *jointless-2* (*j-2*), have been discovered that completely suppress the formation of flower and fruit pedicel AZ. In the in vitro scrutiny, we found a dominant mutant (*1941-MM*) initially selected by increased leaflet lobing (Fig. 1l). In greenhouse-grown plants this feature is accentuated, as leaflets, almost serrated, show deeply indented margins (Fig. 8a-b). Notably, the pedicel abscission zone is activated in flower bud or pre-anthesis stages (Fig. 8c), promoting flower detachment from the inflorescence (Fig. 8d). Thus, the phenotype of *1491-MM* is opposite to that of *jointless* and *jointless-2* and for this reason we named it *Jointmore* (*Jmr*). Since the early detachment of the flowers prevents selfing, we tried to collect pollen to achieve backcross progenies. This likewise proved impossible since in most cases the AZs of flower internal whorls are also activated causing their detachment (Fig. 8g-h). Given the impossibility of obtaining progenies which facilitated a phenotype-*nptII* co-segregation analysis, we studied the expression of the GUS reporter to inquire whether the mutant phenotype could be due to one of the five T-DNA insertions (Fig. 8e). A strong signal was observed in leaf rachis as well as in the stem pith (Fig. 8f). GUS expression was also particularly intense in the pedicel abscission zone from the first floral bud stages until after flower detachment (Fig. 8g-i). In addition, strong reporter expression was detected in the zone where the AZs of the flower internal whorls lie (Fig. 8g-h). Thus, the close association between altered traits and reporter expression in *Jmr* strongly suggests that one of the five T-DNA inserts (Fig. 8e) has tagged a gene involved in abscission processes, promoting its upregulation.

The introgression of *j-2* has been a key factor in the development of tomato cultivars adapted to mechanical harvesting and industrial processing. In addition, *j* and *j-2* allowed initiation of the genetic dissection of the tomato abscission process. *JOINTLESS*, a MADS-box gene of a distinct phylogenetic clade from those functioning in flower whorls, was the first identified [64]. More recently it has been shown that the alteration in another MAD-box gene, *SlMBP21*, accounts for *j-2* phenotype [65]. It has been described that *JOINTLESS* is expressed at very early stages of AZ formation in WT tomato plants [66], somewhat similar to what was observed in the studies on reporter expression in *Jmr*. For this reason, we analyzed the *JOINTLESS* expression in *Jmr*. Results showed that its expression does not change (data not shown), indicating that this is not the gene altered in our mutant.

As mentioned above, our results suggest that, unlike the loss-of-function mutations *j* and *j-2*, *Jmr* is a gain-of-function mutant, probably because one of the T-DNA-insertions promotes the *JMR* upregulation. There is some similarity between the characteristics of *Jmr* and those described in *Petroselinum* (*Pts*), a semi-dominant mutant showing accelerated pedicel and petiole abscission. The leaf shape in the Galapagean tomatoes results from a single-nucleotide deletion in the promoter of the *PETROSELINUM* (*PTS*), a *KNOTTED1-LIKE HOMEOBOX* (*KD1*) gene up-regulated in both leaf and flower abscission zones [67, 68]. The total infertility of *Jmr* prevented an allelism test with *Pts*, although it is unlikely that both mutants are altered in the same gene, since the architecture of the *Jmr* leaf (Fig. 8a) does not show the complexity of *Pts* (see Fig. 1c, reference [67]) and the flowers never reach the anthesis stage, unlike that described in *Pts* (see Additional file 1: Figure S1e; reference [68]. Actually, in spite of the different species and organs involved, the extension of the floral abscission process in *Jmr* is more similar to the seed-shattering trait in US weedy rice [69] or, alternatively, to the effect of constitutive overexpression of *INFLORESCENCE DEFICIENT IN ABSCISSION* (*IDA*) on early abscission of floral organs in Arabidopsis [70].

Abscission processes have been mainly studied in mutants with inhibition of AZ differentiation in the organ or suppression of abscission processes in AZ cells [71]. The discovery of a new tomato mutant with an early and generalized activation of floral abscission could provide a different perspective and shed further light on this process. It has also been proposed that the regulation of abscission involves many of the same regulators that function in the determination of meristematic cells [65, 72–74]. Interestingly the alteration of some of these genes also promotes changes in leaf development (e.g. *GOBLET*; [65]), perhaps through their function in the blastozone. We expect future studies with *Jointmore* to bring new insights into the complex networks regulating all these developmental processes.

### Identification of the gene tagged in a mutant altered in leaf and fruit development

**Fig. 9:**
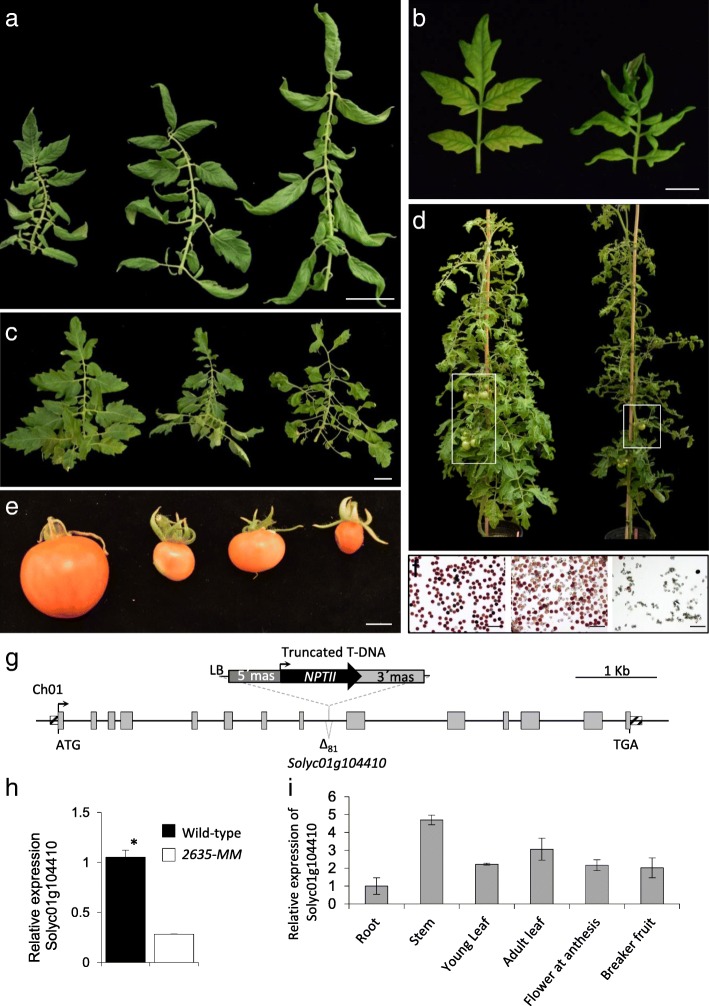
Vegetative and reproductive development and molecular characterization of the gene tagged in the mutant *Tomato ungainly leaves* (*Tul*). **a** Clonal replicates of T0 plants develop leaves with a variable number of primary and secondary leaflets usually folded towards the abaxial side. **b** Unlike the WT (left) the basal leaves of plants cultivated in vitro of the mutant *Tul* (right) also exhibit a slight folding. **c** Adult leaves of WT (left) and mutant plants (center and right). Note the irregular and ungainly appearance of the mutant leaves. **d** Adult plants of WT (left) and mutant *Tul* (right). **e** Variability in the fruits of the *Tul* mutant. **f** Pollen from WT (left) and mutant plants (centre and right) stained with 0.5% TTC. Note the smaller amount of viable pollen, or even its total absence, in the anthers of mutant flowers. **g** Schematic representation of T-DNA insertion in the *Solyc01g104410* gene. Coding sequences and 5′ and 3′ untranslated regions of the *Solyc01g104410* gene are depicted as grey and striped boxes, respectively. The truncated T-DNA insertion contains the left border (LB) and the *NEOMYCIN PHOSPHOTRANSFERASE II* (*NPTII*) gene controlled by the promoter and terminator of the manopine synthase (5’mas and 3’mas, respectively. **h** Relative expression of *Solyc01g104410* gene in wild-type and *Tul* mutant plants. Asterisk denotes significant differences at *P* < 0.05. **g** Spatial expression pattern of *Solyc01g104410* gene in wild-type tissues. Bars (**a** and **c**) = 5 cm. Bar (**b** and **f**) =1 cm. Bar (**e**) = 100 μm

Co-segregation analyses showed the existence of an association between a T-DNA insert and the mutant phenotype of *2635-MM* line (named *Tomato ungainly leaves*, *Tul*). The characterization of several clonal replicates of the T0 plant, as well as of the T1 progeny, revealed that in mutant plants the young leaves have a variable number of primary and secondary leaflets that are usually folded towards the abaxial side. (Fig. 9a, b). In adult leaves the number and distribution of leaflets is extremely irregular. In addition, the great variability in the length of the petiolules connecting the rachis with the leaflet causes a characteristic ungainly aspect of adult leaves (Fig. 9c, d). Fruit development also showed a great variability both in the same plant and in different mutant plants. Some fruits developed seeds and reached a size similar to WT, while most were smaller and seedless (Fig. 9e). This irregularity in fruit development contrasted with the normality of the mutant’s flowers, which morphologically did not differ from the WT. Despite this, a thorough analysis revealed a smaller amount of viable pollen, or even its total absence, in the anthers of mutant flowers (Fig. 9f). In a recent study we characterized a tomato mutant (*pollen deficient1*, *pod1*), which also displayed a significant reduction of pollen viability and yielded parthenocarpic fruits [75]. We also reported that the loss-of-function of *MEDIATOR COMPLEX SUBUNIT 18* (*POD1*/*SlMED18*) is responsible for the alterations in reproductive development observed in *pod1*. MED18 is a subunit of the MEDIATOR COMPLEX that binds RNA Polymerase II, a conserved transcriptional regulatory complex of class II genes in eukaryotes [76, 77]. The mutant *pod1* also showed a change in leaf size [75], suggesting that *POD1* could play an additional role in leaf development. In fact, it has recently been observed that the silencing of *POD1* restricts both the elongation of the stem internodes and the expansion of leaf blade [78]. Despite the analogies between *pod1* and *Tul* (lower pollen viability and development of parthenocarpic fruits), both mutants present important differences in leaf morphology (compare Fig. 9c with Fig. 1a reference [75]) as well as in leaf and fruit development (regular in *pod1* and completely irregular in *Tul*). This suggested that the *TUL* gene should be different from *POD1*.

To identify the gene tagged in the *Tul* mutant, anchor-PCR assays were performed to clone the genomic regions flanking the T-DNA insertion. Results revealed that T-DNA was located on chromosome 01 (at position 92,783,095 bp; SL2.50 tomato genome version), in the eighth intron between exons 8 and 9 of the *Solyc01g104410* gene, which codes for a Sterol 3-beta-glucosyltransferase. Results also showed that the *Tul* mutant bore a truncated T-DNA insertion, which underwent some rearrangements during the insertional process as the right border and the *uidA* reporter gene were removed (Fig. 9g). The effects of T-DNA integration on gene expression were determined by quantitative RT-PCR, which showed that the *Solyc01g104410* gene was significantly down-regulated in mutant plants as compared to wild-type ones (Fig. 9g). The analysis of the spatial expression pattern of the *Solyc01g104410* gene in wild-type plants indicated that transcripts were mainly accumulated in stem and adult leaf, although it was also expressed in flowers and fruits (Fig. 9i). Hence, results suggest that the abnormal leaves and alteration of fruit development in *Tul* mutant plants might be due to altered steryl glycoside biosynthesis caused by the lack-off-function of the *Solyc01g104410* gene. In agreement with this hypothesis, genetic manipulation of the sterol biosynthetic pathway in *Nicotiana benthamiana* results in a growth reduction phenotype [79]. In addition, it is worth stating that a high content of steryl glycosides is accumulated in plants of the genus *Solanum,* unlike most plant species where steryl glycosides are generally minor components of the total sterol fraction. Thus, in the case of tomato leaves, the steryl glycosides represent 83% of total sterols [80]. Even though the biological significance of the high content of steryl glycosides in *Solanum* species is unknown, it has been suggested that they play a role in protecting cell membrane integrity, reducing the disruptive effect of steroidal glycoalkaloids which are present at high levels in plants of the genus *Solanum* [81–83].

## Conclusions

To implement a faster but reliable scrutiny method for identifying tomato mutants altered in leaf morphology we addressed the screening in vitro of 971 T-DNA lines. Putative mutants selected in vitro were evaluated in the greenhouse. The comparison of results in both conditions indicated a general phenotypic correspondence, showing that in vitro culture is a reliable system for finding mutants altered in size, shape, lobing, wrinkling, bending and leaf architecture. Apart from providing homogeneous conditions around the year, the main advantage of screening in vitro lies in the enormous saving of time and space. This method also facilitated the study of root development, which, together with grafting experiments, allowed assessment of the relative effect of a mutation on the aerial part and root system. Studies on the association between phenotype and *nptII* gene expression showed co-segregation in two lines with *P* > 99%. We also took advantage of the use of an enhancer trap for identifying gain-of-function mutants through reporter expression analysis. Two new tomato mutants putatively altered in synthesis or perception of brassinosteroids were identified. The extreme phenotype of one of them was partially restored upon treatment with brassinolide. With the other, the close association between phenotypic changes and reporter expression pattern strongly suggested that the altered gene is T-DNA tagged. Root development was studied in a new *wire-like* mutant, an aspect that to the best of our knowledge has not been described in other *wiry* mutants. Primary root development was unaffected but lateral root growth was significantly delayed in both embryo-derived root and adventitious root system. We also found the first tomato mutant with helical growth. Unlike what happens with some previously described mutants of Arabidopsis, in which the helical growth is associated with a reduced anisotropic growth, in our mutant the phenotype seems to be due to the opposite mechanism, that is, an increase in cellular anisotropy. The characterization of other mutants revealed new connections between leaf development and some agronomic traits. For instance, the study of a tomato mutant showing curvature in almost all vegetative and reproductive organs revealed a possible functional link between leaf wrinkling, cuticle roughness and tendency to fruit cracking. The discovery of another mutant with extreme leaf bending and restricted axillary growth provided additional evidence on common mechanisms regulating both developmental processes. Studies with a gain-of function mutant selected by irregular distribution of leaflets suggested a new functional link between leaf development and fruit setting. Similarly, a close association between phenotype and reporter expression pattern was found in a new tomato mutant showing an early and generalized activation of external and internal floral AZs. Abscission processes have until now been studied in mutants with inhibition or suppression of AZs. The discovery of this mutant could provide a different perspective to gain new insights into this process. Finally, Anchor-PCR assays showed that the gene tagged in the mutant *2635-MM* encodes for a Sterol 3-beta-glucosyltransferase. Quantitative RT-PCR analysis suggested that that the abnormal leaves and alteration of fruit development in mutant plants might be due to altered steryl glycoside biosynthesis caused by the lack-off-function of this gene. As far as we know, this is the first time that the possible implication of this gene in tomato leaf and fruit development is reported.

## Methods

### Characteristics of T-DNA lines from tomato and wild related species

Several collections of T-DNA lines were generated from the tomato cultivar ‘Moneymaker’ (accession LA2706, Tomato Genetics Resource Center, http://tgrc.ucdavis.edu/), the tomato line ‘P73’, and the accession ‘PE47’ of *Solanum pennellii* (kindly provided by Dr. Rafael Fernández Muñoz, IHSM, La Mayora, CSIC, Málaga Spain) using the methods previously described [46, 84]. The enhancer trap vector used for transformation was pD991 (kindly supplied by Dr. Thomas Jack; Department of Biological Sciences, Dartmouth College, USA), which has been previously described [85]. Basically, the pD991 enhancer trap vector includes, at the 5′ end and close to the right border (RB), the *uidA* gene coding for GUS enzyme preceded by a minimal promoter, the latter being insufficient to drive GUS expression. In addition, this vector contains the selection marker gene *neomycin phosphotransferase II* (*nptII*) near the left border (LB) at the 3′ end of the T-DNA, controlled by the 5′ mannopine synthase (*mas*) promoter and the 3′ *mas* terminator.

### Screening in vitro of tomato mutants altered in leaf development

971 tomato T-DNA lines (‘Monemaker’ and ‘P73’) were screened for changes in leaf development in vitro as described below. In addition, during the scrutiny of a collection of *S. pennellii* T-DNA lines aimed at identifying mutants with changes in salinity tolerance, some mutants were detected that were not affected in salt tolerance but in leaf development. Due to their special interest, two of these mutants were incorporated into the present study. In the case of T-DNA lines with no problems of fertility, the screening in vitro was carried out in both seedlings and axenic plants from T1 progenies. Seeds were surface sterilized by immersion in 12.5% commercial bleach (a sodium hypochlorite solution equivalent to 50 g L^− 1^ of active chlorine) for 20 min followed by three rinses with sterile distilled water, and sown aseptically on MG medium consisting of MS salt solution [86] supplemented with 1% sucrose and 0.8% Agar (Bacteriológico Europeo, Pronadisa) in 85 × 120 mm culture vessels covered with translucent plastic plugs. Five seedlings were cultivated in each culture vessel. In order to evaluate axenic plants, shoot-apex and axillary bud explants were cut off and transferred individually to culture vessels of 55 × 150 mm with MB3 medium [87]. In the case of T-DNA lines with problems of fertility several clonal replicates for each T0 line were obtained by culturing shoot-apex and axillary buds in the recipients and culture medium mentioned above. The pH of all culture media was adjusted to 5.7 before autoclaving at 121 °C for 25 min. The incubation of plant material was carried out in a growth chamber at 25 ± 1,5 °C under a photoperiod of 16 h light/8 h dark and a photon fluence rate of 90 μmol m^− 2^ s^− 1^ (Grolux, Sylvania, fluorescent tubes). Alterations in leaf traits (e.g. size, shape, architecture, lobing, wrinkling, bending) were evaluated at 15, 20 and 30 days of incubation. Upon detecting a putative mutant altered in leaf development three additional experiments were performed in order to confirm the reproducibility of the phenotype. When an anomaly in the root system was detected an additional study in large test tubes (36 × 250 mm) was carried out. Primary and lateral root development was studied in seedlings grown on MG medium for 30 days. To study adventitious rooting shoot apexes were subcultured in MB3 culture medium and incubated for 45 days. The detection of mutants with changes in the type or number of trichomes was performed by observing the abaxial and adaxial sides of the leaf blade on a stereoscopic magnifying glass. The change in the number of trichomes was evaluated qualitatively by comparison with the WT. The classification of the type of trichomes affected in tomato or *Solanum pennellii* mutants was based on the review by Simmons and Gurr [28].

### Characterization in vivo of tomato mutants altered in leaf development

All evaluations *in planta* were conducted in a controlled greenhouse environment, under the following conditions: long-day photoperiod (16 h of natural light supplemented with Osram lamps Powerstar HQI-BT, 400 W), temperature fixed at 24 °C during the day and 18 °C at night, and automatic fertigation. Plants were daily irrigated with Hoagland’s nutrient solution [41] in 6 L pots with a mixture of peat:vermiculite (1:1 *v*/v). To determine the leaf development parameters of each mutant, the fifth leaf was generally used. However, younger leaves (2° - 3°) and adult leaves were also evaluated. In general, the number and size of primary and intercalary leaflets were assessed. In some mutants, the length of the rachis and/or petiolules was also evaluated. In more complex leaves, the presence of secondary and tertiary leaflets was evaluated qualitatively. Similarly, the degree of wrinkling, bending, lobing or indentation of the leaflets was qualitatively estimated. To study other vegetative traits, parameters related to shoot apical meristem development, number and length of phytomers of the initial segment (before the first inflorescence) and that of the successive sympodial segments up to the seventh inflorescence, were all evaluated. As regards reproductive development the architecture of inflorescence and that of the four flower whorls (sepals, petals, stamens and carpel) as well as fruit size and shape, skin color and seed number were analyzed.

### Estimation of the number of T-DNA inserts

In the case of fertile mutants, a segregation analysis was performed in T1 progenies to estimate the number of inserts carrying a functional *nptII* marker gene. Cotyledons from 7-day-old seedlings were cut off and portions of 2 mm width along the proximal and distal edges were removed before they were placed in 9 cm Petri dishes with the abaxial side towards the culture medium. The culture medium consisted of MB3 basal medium supplemented with 4 mg L^− 1^ indole-3-acetic acid (IAA), 4 mg L^− 1^ 6-furfurylaminopurine (kinetin, K), 1 mg L^− 1^ trans-zeatin (Z) and 100 mg l^− 1^ kanamycin. In mutants with infertility problems, the number of inserts was determined by Southern blot following the methods previously described [13, 68].

### Co-segregation analysis

In mutants with no problems of fertility and with one T-DNA insert bearing a functional *nptII* marker gene, co-segregation analysis was performed in vitro by studying the association between the mutant phenotype and the *nptII* gene expression. In the case of a recessive mutation, the comparison of the genetic models corresponding to the existence, or not, of co-segregation between the mutant phenotype and the T-DNA insert with a functional *nptII* gene indicate that, in the second case, the probability associated to the appearance of a mutant plant (M) sensitive to kanamycin (kanS) equals 1/16. Therefore, if 47 plants are analyzed and no M-kanS plant is detected, the existence of cosegregation can be accepted with a probability of 95% (or 99% if 72 plants are evaluated). Similarly, for a dominant mutation not due to a T-DNA insert, the probability of finding a M-kanS plant would be 3/16. Therefore, if 15 plants are analyzed and no M-kanS is detected, the existence of co-segregation can be accepted with a 95% probability (or 99% if 23 plants are analyzed). In the case of mutants with infertility problems, we used a different approach to assess whether a T-DNA insert could have tagged the mutant allele. The strategy was based on the use was of an enhancer trap, since, if the T-DNA is inserted in the appropriate direction near or within an endogenous gene, the reporter expression pattern can mimic that of the latter [22]. This offers the possibility of identifying gain-of-function mutants in which there is a correspondence between the phenotypic changes and the reporter expression pattern in certain organs. In addition, the enhancer trap acts as a dominant element, whose expression pattern can be detected in hemizygous state [85]. GUS histochemical assays were carried out as described by Atarés et al. [46]. Basically, the samples of different tissues of T0 plants were collected and placed in GUS staining solution [100 mM sodium phosphate at pH 7.0, 0.1% Triton X-100, 10 mM ethylenediaminetetraacetic acid (EDTA), 0.5 mg/mL X-Gluc, 0.5 mM potassium ferricyanide, 0.5 mM potassium ferrocyanide and 20% methanol] and incubated at 37 °C for 20–24 h. Subsequently, the GUS-stained tissues were washed with 70% ethanol for chlorophyll removal and examined under a zoom stereomicroscope (MZFLIII, Leica). Three replicates of each sample were analysed.

### Histology techniques

Plant tissue was fixed in FAE (50% [v/v] ethanol, 5% [v/v] formaldehyde, 10% [v/v] acetic acid) and stored in 70% [v/v] ethanol. Subsequently, tissues were dehydrated in 100% [v/v] ethanol and embedded in paraffin (Paraplast Plus) blocks using plastic containers. Sections (8 μm thick) of material were cut with a Leica RM2025 microtome and stained with toluidine blue: 2–5 min in 0,05% (*w*/*v*) toluidine blue and rinsed with water. The samples were observed with a Leica MZZ16F light microscope (Leica Microsystems, Wetzlar, Germany).

### Cloning of T-DNA flanking sequences and expression analysis

Sequences flanking the *2635-MM* T-DNA insertion were cloned by a modified anchor-PCR according to the protocol previously described [23]. The cloned sequences were compared with SGN Database (http://solgenomics.net/) to assign the T-DNA insertion site on tomato genome. Expression analyses were performed with three biological and two technical replicates. Total RNA was isolated using TRIZOL (Invitrogen, Carlsbad, CA, USA) following the manufacturer’s instructions. cDNA was synthesized from 500 ng of total RNA using the M-MuLV reverse transcriptase (Fermentas Life Sciences, Hanover, MD, USA) with a mixture of random hexamer and oligo(dT)18 primers. Quantitative RT-PCRs were performed with the SYBR Green PCR Master Mix (Applied Biosystems, Foster City, CA, USA) kit using the 7300 Real-Time PCR System (Applied Biosystems). The housekeeping *Ubiquitine3* (*Solyc01g056940*) gene was used as control in all gene expression analyses. Specific primer pairs for *Solyc01g056940* (forward 5′- CACACTTCACTTGGTCTTGCGT - 3′ and reverse 5′- TAGTCTTTCCGGTGAGAGTCTTCA - 3′ primers) and *Solyc01g104410* (forward 5′- GCTGGTGTTCCTCAGGTGATATG - 3′ and reverse 5′- GCAACACCGAGCCAATACATCC - 3′ primers) genes were designed. Results were expressed using the ΔΔCt calculation method [88] in arbitrary units by comparison with a data point from the wild-type samples.

## Additional files

Additional file 1: **Figure S1.** Morphology of the leaves of greenhouse-grown plants of tomato cv ‘P73’ and some dominant mutants. **a** Fifth leaf of a tomato plant cv. ‘P73’. **b-c** The mutants *816-P73* (**b**) and *740-P73* (**c**) have darker, less lobed leaves. **d-f** The mutants *136-P73* (**d**), *605-P73* (**e**) and *14-P73* (**f**) develop leaflets of different morphology. **g** The mutant *630-P73* develops small leaves with irregular arrangement of leaflets. **h** The mutant *860-P73* has more complex leaves with smaller leaflets. Bar = 1 cm. (PPTX 922 kb)

Additional file 2: **Table S1.** Phenotype inheritance in tomato mutants altered in leaf development. (DOCX 20 kb)

Additional file 3: **Figure S2.** Identification in vitro of mutants altered in trichome development. **a** Most type I trichomes of the mutant *744-P73* have abnormal terminal cells. **b** The leaves of *1491-MM* have fewer type I trichomes. **c** The leaves of *912-P73* have fewer trichomes of types I and VI. **d** The leaves (right, up) and stem (right, down) of the mutant *4728-SP* have a greater number of trichomes with respect to *Solanum pennellii* wild-type plants (left, up and down). Bar = 1 mm. (PPTX 2580 kb)

Additional file 4: **Table S2.** Number of T-DNA inserts with a functional nptII marker gene in tomato mutants altered in leaf development. (DOCX 19 kb)

Additional file 5: **Table S3.** Co-segregation analysis between phenotype and a T-DNA insert with a functional nptII gene in mutants altered in leaf development. (DOCX 14 kb)

Additional file 6: **Figure S3.** Vegetative and reproductive development of the tomato mutant *wiry-like-150* (*wl-150*). **a** Basal leaves of *wl-150* have a certain degree of leaf blade expansion, while the following exhibit shoestring shape. **b** The inflorescence of *wl-150* (right) is more branched than that of wild-type ‘P73’ plants (left). Note that the inflorescences of *wl-150* also alternate vegetative and reproductive traits. **c** Flowers of *wl-150* have thread-like sepals and petals as well as an open anther cone. **d** The mutant *wl-150* is partially fertile since it develops fruits ranging from small seedless (right, up) to others of normal size with some seeds (right, down) similar to that of wild-type ‘P73’ (fruit on the left). Bar = 1 cm. (PPTX 492 kb)

Additional file 7: **Figure S4.** Vegetative development of the tomato mutant *Reduced growth of lateral branches* (*Rgb*). **a-b** Leaves of *Rgb* mutant (**b**) are about a quarter the size of WT (**a**) and leaflets are completely bent in on themselves (**b**). **c-d** The lateral branches of *Rgb* mutant (**d**) are much shorter than those of WT (**c**). **e** The *Rgb* plant reaches a similar height to WT but, due to leaf bending and the development of multiple short lateral branches, it seems as if vegetative structures were compressed around the stem. Bar = 5 cm. (PPTX 785 kb)

Additional file 8: **Figure S5.** Vegetative and reproductive development of the tomato mutant *necrotic and small leaflets* (*nsl*). **a**. Mutant *nsl* seedlings (right) develop smaller leaves than WT (left). **b**. In the mutant *nsl* the leaves of shoot apex-derived plants have small necrotic spots (right), which does not occur in the WT (left). **c**. In *nsl* greenhouse-grown plants adult leaves have a great number of small leaflets that exhibit necrotic spots in the leaf blade. **d**. In *nsl* mutant plants a decrease in quantitative parameters related to the number of fruits per inflorescence, fruit set rate and fruit size is observed. Bar = 1 cm. (PPTX 627 kb)

Additional file 9: **Figure S6.** Flowering time in plants of the mutant *Altered in all reproductive traits* (*Art*). **a**. Number of phytomers up to the first inflorescence (0–1) and between inflorescences (1–2, 2–3, …). **b** Length from base up to the first inflorescence (0–1) and between inflorescences (1–2, 2–3, …). (PPTX 63 kb)

## Abbreviations

AZs: Abscission zones
