# Supplementary figures and images for: Phenotypic and genetic characterization of tomato mutants provides new insights into leaf development and its relationship to agronomic traits
Source: BMC Plant Biol. 2019 Apr 15;19:141. doi: 10.1186/s12870-019-1735-9 (PMC6466659; doi:10.1186/s12870-019-1735-9)

## Slide 1
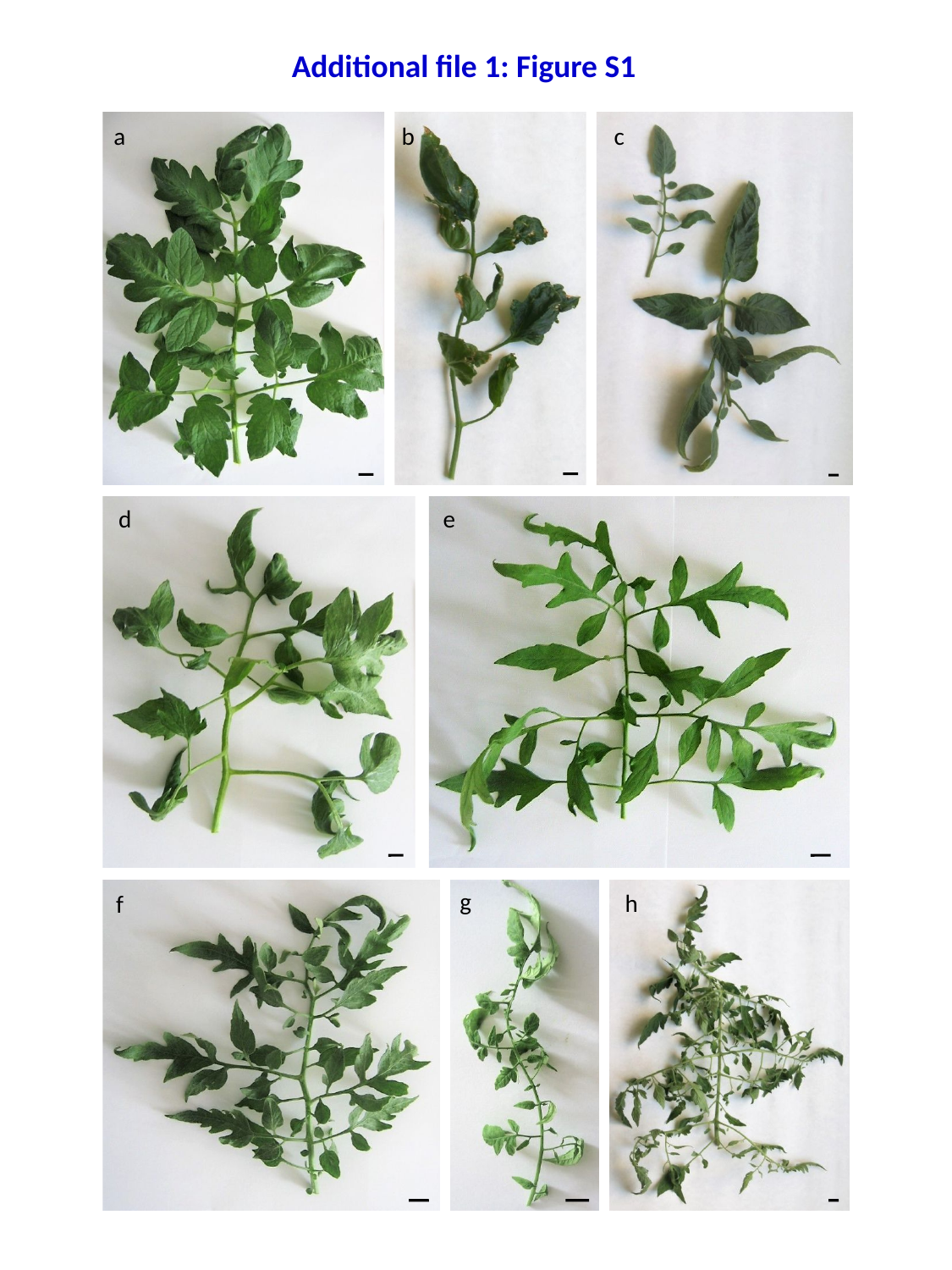

Additional file 1: Figure S1
a
b
c
d
e
g
h
f

Supplement: Supplementary file 1 — Figure S1. Morphology of the leaves of greenhouse-grown plants of tomato cv ‘P73’ and some dominant mutants. a Fifth leaf of a tomato plant cv. ‘P73’. b-c The mutants 816-P73 (b) and 740-P73 (c) have darker, less lobed leaves. d-f The mutants 136-P73 (d), 605-P73 (e) and 14-P73 (f) develop leaflets of different morphology. g The mutant 630-P73 develops small leaves with irregular arrangement of leaflets. h The mutant 860-P73 has more complex leaves with smaller leaflets. Bar = 1 cm. (PPTX 922 kb) [file 12870_2019_1735_MOESM1_ESM.pptx]

## Slide 1
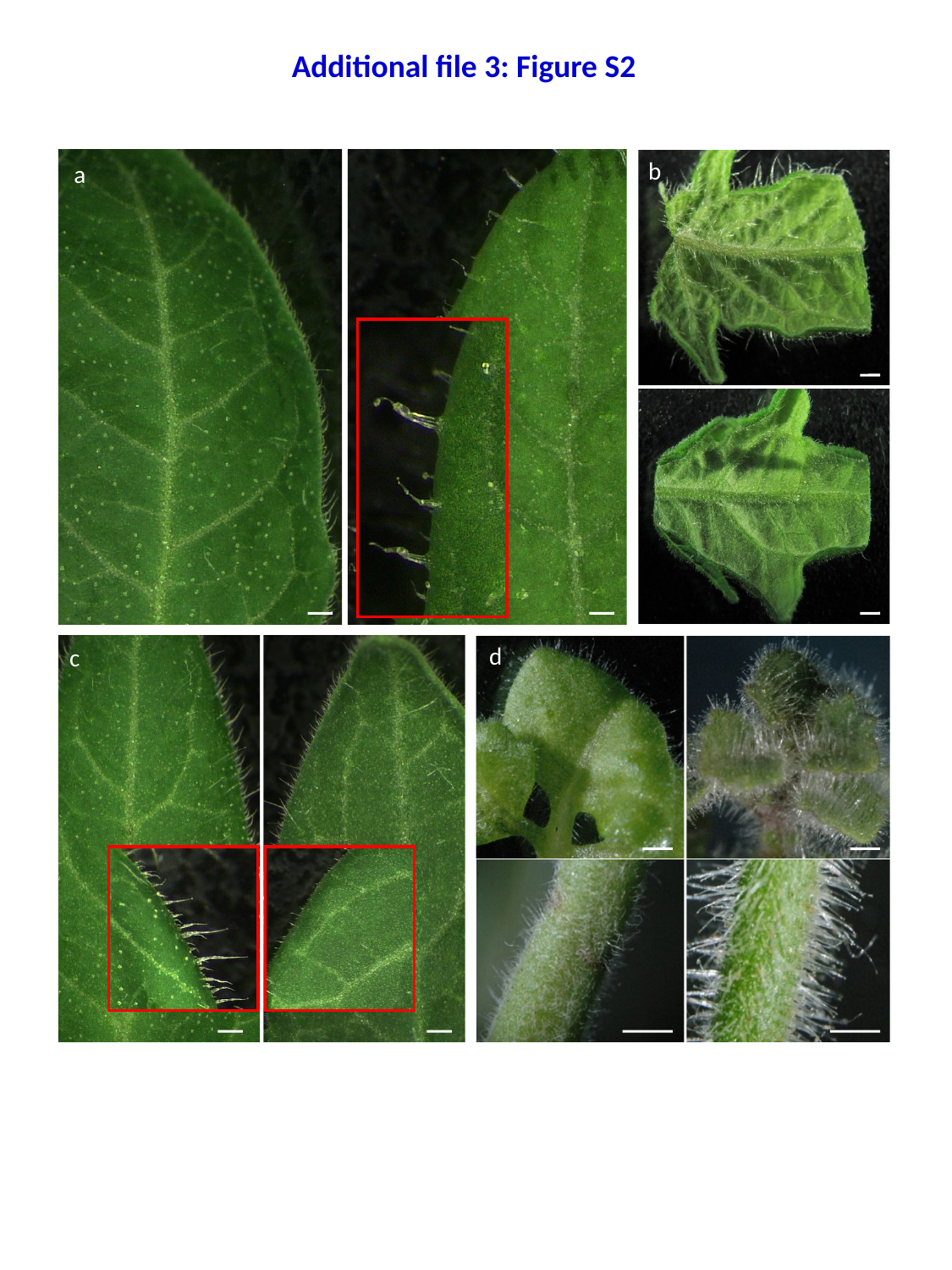

Additional file 3: Figure S2
b
a
d
c

Supplement: Supplementary file 3 — Figure S2. Identification in vitro of mutants altered in trichome development. a Most type I trichomes of the mutant 744-P73 have abnormal terminal cells. b The leaves of 1491-MM have fewer type I trichomes. c The leaves of 912-P73 have fewer trichomes of types I and VI. d The leaves (right, up) and stem (right, down) of the mutant 4728-SP have a greater number of trichomes with respect to Solanum pennellii wild-type plants (left, up and down). Bar = 1 mm. (PPTX 2580 kb) [file 12870_2019_1735_MOESM3_ESM.pptx]

## Slide 1
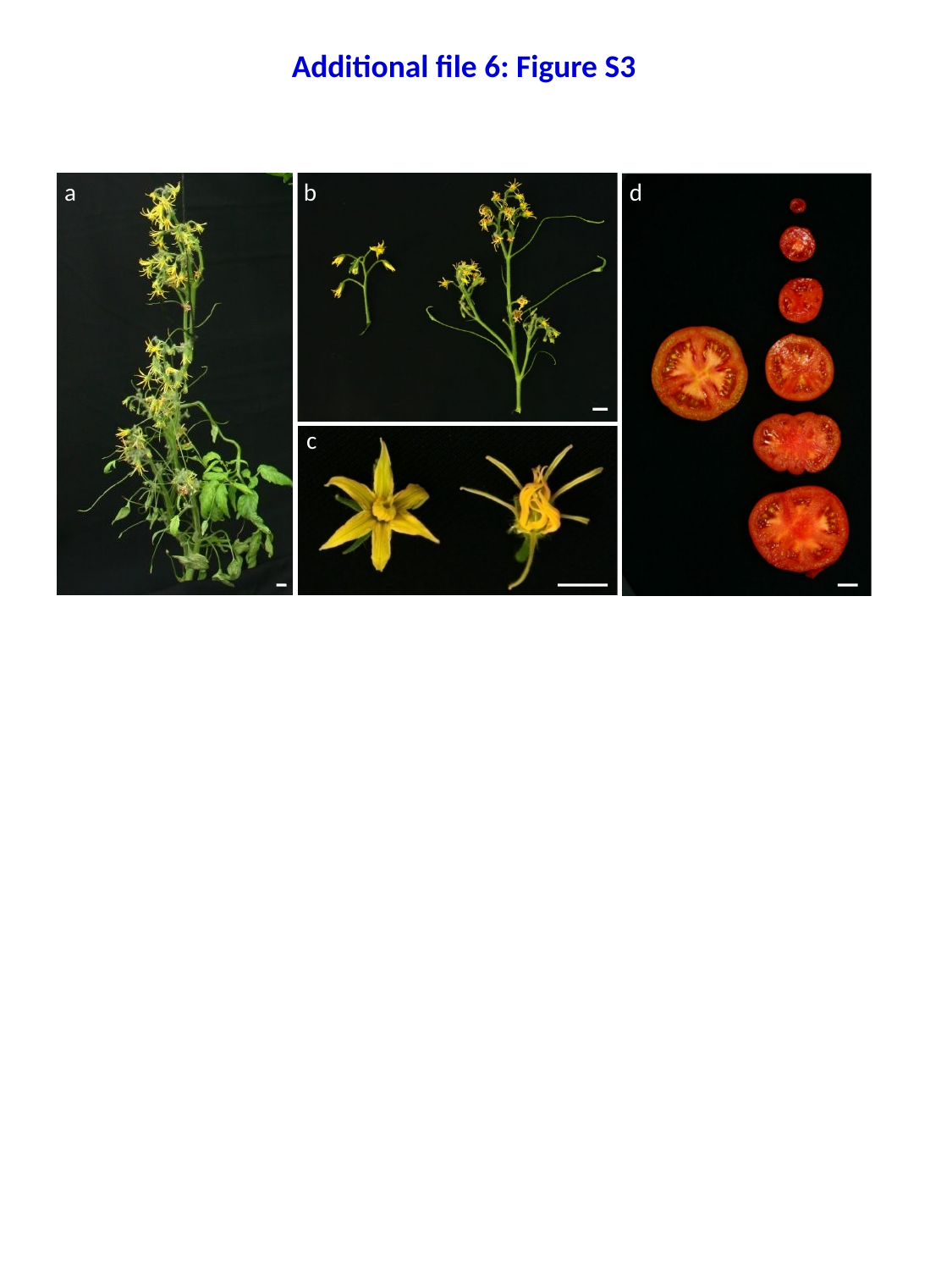

Additional file 6: Figure S3
a
b
d
c

Supplement: Supplementary file 6 — Figure S3. Vegetative and reproductive development of the tomato mutant wiry-like-150 (wl-150). a Basal leaves of wl-150 have a certain degree of leaf blade expansion, while the following exhibit shoestring shape. b The inflorescence of wl-150 (right) is more branched than that of wild-type ‘P73’ plants (left). Note that the inflorescences of wl-150 also alternate vegetative and reproductive traits. c Flowers of wl-150 have thread-like sepals and petals as well as an open anther cone. d The mutant wl-150 is partially fertile since it develops fruits ranging from small seedless (right, up) to others of normal size with some seeds (right, down) similar to that of wild-type ‘P73’ (fruit on the left). Bar = 1 cm. (PPTX 492 kb) [file 12870_2019_1735_MOESM6_ESM.pptx]

## Slide 1
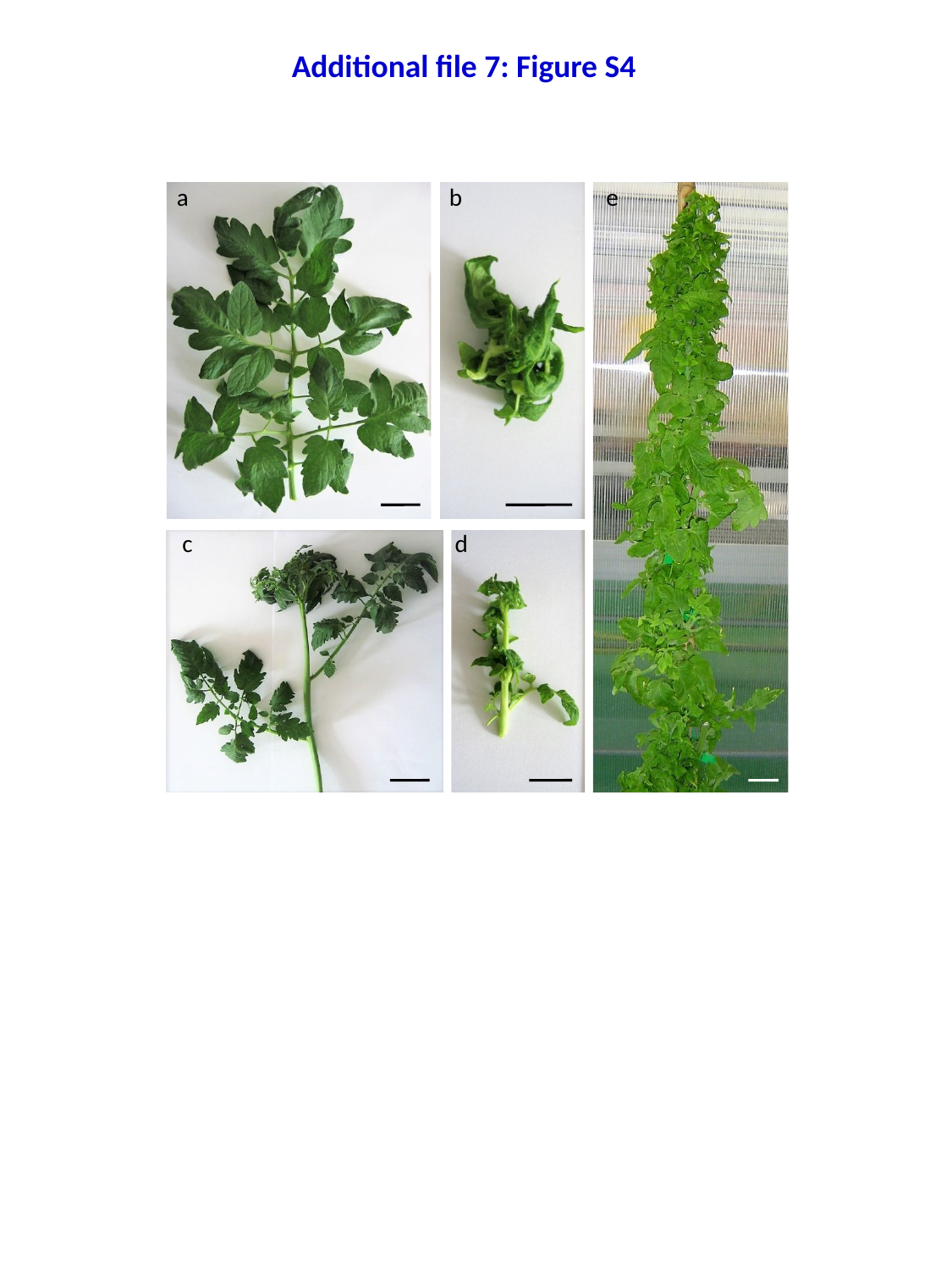

Additional file 7: Figure S4
a
b
e
c
d

Supplement: Supplementary file 7 — Figure S4. Vegetative development of the tomato mutant Reduced growth of lateral branches (Rgb). a-b Leaves of Rgb mutant (b) are about a quarter the size of WT (a) and leaflets are completely bent in on themselves (b). c-d The lateral branches of Rgb mutant (d) are much shorter than those of WT (c). e The Rgb plant reaches a similar height to WT but, due to leaf bending and the development of multiple short lateral branches, it seems as if vegetative structures were compressed around the stem. Bar = 5 cm. (PPTX 785 kb) [file 12870_2019_1735_MOESM7_ESM.pptx]
