# Supplementary material for: Phenotypic and genetic characterization of tomato mutants provides new insights into leaf development and its relationship to agronomic traits
Source: BMC Plant Biol. 2019 Apr 15;19:141. doi: 10.1186/s12870-019-1735-9 (PMC6466659; doi:10.1186/s12870-019-1735-9)
Supplement: Supplementary file 2 — Table S1. Phenotype inheritance in tomato mutants altered in leaf development. (DOCX 20 kb) [file 12870_2019_1735_MOESM2_ESM.docx]

**Additional file 2: Table S1. Phenotype inheritance in tomato mutants altered in leaf development**.

Recessive

|  | Observed (expected) segregation | | χ^2 b^ |  |
| --- | --- | --- | --- | --- |
| Mutant ^a^ | Wild-type | Mutant |  |  |
|  |  |  |  |  |
| *150-P73* | 35 (40.5) | 19 (13.5) | 2,99 | P = 0,084 |
|  |  |  |  |  |
| *272-P73* | 68 (54) | 4 (18) | 14,52 | P = 0,000 |
|  |  |  |  |  |
| *700-P73* | 51 (47.25) | 12 (15.75) | 1,19 | P = 0,017 |
|  |  |  |  |  |
| *1381-P73* | 141 (144) | 51 (48) | 0,25 | P = 0,617 |
|  |  |  |  |  |
| *1425-MM* | 93 (90) | 27 (30) | 0,40 | P = 0,527 |
|  |  |  |  |  |
| *1458-MM* | 75 (73,5) | 23 (24,5) | 0,12 | P = 0,726 |
|  |  |  |  |  |
| *1527-MM* | 58 (58,5) | 20 (19,5) | 0,02 | P = 0,896 |
|  |  |  |  |  |
| *2059-MM* | 65 (62) | 18 (21) | 0,90 | P = 0,449 |
|  |  |  |  |  |
| *2489-MM* | 45 (42,7) | 12 (14,3) | 0,47 | P = 0,482 |
|  |  |  |  |  |
| *2733-MM* | 40 (33,7) | 5 (11,3) | 4,63 | P = 0,030 |
|  |  |  |  |  |
| *2742-MM* | 75 (70,5) | 19 (23,5) | 1,15 | P = 0,284 |
|  |  |  |  |  |
| (a) The denomination of the T-DNA lines is explained in Table 1  (b) The χ^2^ calculated value represents the fit of the data to an expected ratio 3 WT : 1 mutant. | | | | |

Dominant

|  | Observed (expected) segregation ^a^ | | χ^2 a^ |  |
| --- | --- | --- | --- | --- |
| Mutant | Wild-type | Mutant |  |  |
|  |  |  |  |  |
| *2635 MM* | 16 (11,5) | 30 (34,5) | 2,35 | P = 0,125 |
|  |  |  |  |  |
|  |  |  |  |  |
| (a) The denomination of the T-DNA lines is explained in Table 1  (b) The χ^2^ calculated value represents the fit of the data to an expected ratio 1 WT : 3 mutant. | | | | |
