# Supplementary material for: Phenotypic and genetic characterization of tomato mutants provides new insights into leaf development and its relationship to agronomic traits
Source: BMC Plant Biol. 2019 Apr 15;19:141. doi: 10.1186/s12870-019-1735-9 (PMC6466659; doi:10.1186/s12870-019-1735-9)
Supplement: Supplementary file 4 — Table S2. Number of T-DNA inserts with a functional nptII marker gene in tomato mutants altered in leaf development. (DOCX 19 kb) [file 12870_2019_1735_MOESM4_ESM.docx]

**Additional file 4: Table S2. Number of T-DNA inserts with a functional *nptII* marker gene in tomato mutants altered in leaf development**.

| Mutant ^a^ | Expected  segregation ^b^ | Observed (expected) data | | χ^2 c^ | P ^d^ |
| --- | --- | --- | --- | --- | --- |
|  |  | Kan Resistant | Kan Sensitive |  |  |
|  |  |  |  |  |  |
| *272 P73* | 3 R : 1 S | 48 (54) | 24 (18) | 2,67 | P = 0,102 |
|  |  |  |  |  |  |
| *700 P73* | 3 R : 1 S | 38 (38,25) | 13 (12,75) | 0,01 | P = 0,936 |
|  |  |  |  |  |  |
| *1381 P73* | 3 R : 1 S | 61 (56,5) | 17 (19,5) | 0,43 | P = 0,410 |
|  |  |  |  |  |  |
| *1425 MM* | 3 R : 1 S  15 R : 1 S | 48 (37,5)  48 (46,9) | 2 (12,5)  2 (1,1) | 11,76  0,43 | P = 0,001  P = 0,383 |
|  |  |  |  |  |  |
| *1458 MM* | 3 R : 1 S | 51 (56,25) | 24 (18,75) | 1,96 | P = 0,162 |
|  |  |  |  |  |  |
| *1527 MM* | 3 R : 1 S  15 R : 1 S  63 R : 1 S | 41 (31,5)  41 (39,4)  41 (41,3) | 1 (10,5)  1 (2,6)  1 (0,7) | 11,46  1,07  0,18 | P = 0,001  P = 0,306  P = 0,718 |
|  |  |  |  |  |  |
| *2059 MM* | 3 R : 1 S | 52 (53) | 19 (18) | 0,12 | P = 0,785 |
|  |  |  |  |  |  |
| *2489 MM* | 3 R : 1 S  15 R : 1 S | 55 (42,75)  55 (53,4) | 2 (14,25)  2 (3,6) | 14,04  0,73 | P = 0,000  P = 0,384 |
|  |  |  |  |  |  |
| *2635 MM* | 3 R : 1 S | 37 (35,2) | 10 (11,8) | 0,35 | P = 0,545 |
|  |  |  |  |  |  |
| *2733 MM* | 3 R : 1 S | 33 (33,7) | 12 (11,3) | 0,07 | P = 0,810 |
|  |  |  |  |  |  |
| *2742 MM* | 3 R : 1 S | 34 (36,75) | 15 (12,25) | 0,82 | P = 0,364 |
|  |  |  |  |  |  |
| (a) The denomination of the T-DNA lines is explained in Table 1.  (b) Expected segregation for one (3 KanR : 1 KanS), two (15 KanR : 1 KanS) or three (63 KanR : 1 KanS) T-DNA inserts with a functional *nptII* gene.  (c) The calculated value represents the fit of the data to the expected segregation.  (d) Probability associated with the corresponding X^2^ value. | | | | | |
