# Supplementary material for: Phenotypic and genetic characterization of tomato mutants provides new insights into leaf development and its relationship to agronomic traits
Source: BMC Plant Biol. 2019 Apr 15;19:141. doi: 10.1186/s12870-019-1735-9 (PMC6466659; doi:10.1186/s12870-019-1735-9)
Supplement: Supplementary file 5 — Table S3. Co-segregation analysis between phenotype and a T-DNA insert with a functional nptII gene in mutants altered in leaf development. (DOCX 14 kb) [file 12870_2019_1735_MOESM5_ESM.docx]

**Additional file 5: Table S3. Co-segregation analysis between phenotype and a T-DNA insert with a functional *nptII* gene in mutants altered in leaf development.**

**Analysis in T1 progenies of recessive mutants with one T-DNA insert**.

|  | Observed segregation ^b^ | | | | | Phenotype–*nptII* association ^c^ |
| --- | --- | --- | --- | --- | --- | --- |
| Mutant ^a^ | WT – R | WT – S | M – R | M – S | Total |  |
|  |  |  |  |  |  |  |
| *272-P73* | 45 | 23 | 3 | 1 | 72 | No co-segregation |
|  |  |  |  |  |  |  |
| *700-P73* | 30 | 8 | 9 | 2 | 49 | No co-segregation |
|  |  |  |  |  |  |  |
| *1381-MM* | 41 | 17 | 20 | 0 | 78 | Co-segregation (P > 99%) |
|  |  |  |  |  |  |  |
| *1458-MM* | 37 | 20 | 14 | 4 | 75 | No co-segregation |
|  |  |  |  |  |  |  |
| *2059-MM* | 39 | 13 | 17 | 2 | 71 | No co-segregation |
|  |  |  |  |  |  |  |
| *2733-MM* | 31 | 11 | 4 | 2 | 48 | No co-segregation |
|  |  |  |  |  |  |  |
| *2742-MM* | 27 | 14 | 7 | 4 | 52 | No co-segregation |
|  |  |  |  |  |  |  |

**Analysis in the T1 progeny of the dominant mutant *2635-MM* with one T-DNA insert**

|  | Observed segregation ^b^ | | | | | Phenotype–*nptII* association ^c^ |
| --- | --- | --- | --- | --- | --- | --- |
| Mutant ^a^ | M – R | M – S | WT – R | WT – S | Total |  |
|  |  |  |  |  |  |  |
| *2635-MM* | 22 | 0 | 0 | 10 | 32 | Co-segregation (P > 99%) |
|  |  |  |  |  |  |  |

(a) See table 1 for denomination of the T-DNA lines. (b) WT: wild-type; M: mutant phenotype; R: kanamycin resistant; S: kanamycin sensitive. (c) The way to carry out the co-segregation analysis to reach a significant conclusion in statistical terms (probability > 99%) is indicated in Methods
