# Supplementary material for: Phenotypic and genetic characterization of tomato mutants provides new insights into leaf development and its relationship to agronomic traits
Source: BMC Plant Biol. 2019 Apr 15;19:141. doi: 10.1186/s12870-019-1735-9 (PMC6466659; doi:10.1186/s12870-019-1735-9)
Supplement: Supplementary file 8 — Figure S5. Vegetative and reproductive development of the tomato mutant necrotic and small leaflets (nsl). a. Mutant nsl seedlings (right) develop smaller leaves than WT (left). b. In the mutant nsl the leaves of shoot apex-derived plants have small necrotic spots (right), which does not occur in the WT (left). c. In nsl greenhouse-grown plants adult leaves have a great number of small leaflets that exhibit necrotic spots in the leaf blade. d. In nsl mutant plants a decrease in quantitative parameters related to the number of fruits per inflorescence, fruit set rate and fruit size is observed. Bar = 1 cm. (PPTX 627 kb) [file 12870_2019_1735_MOESM8_ESM.pptx]

## Slide 1
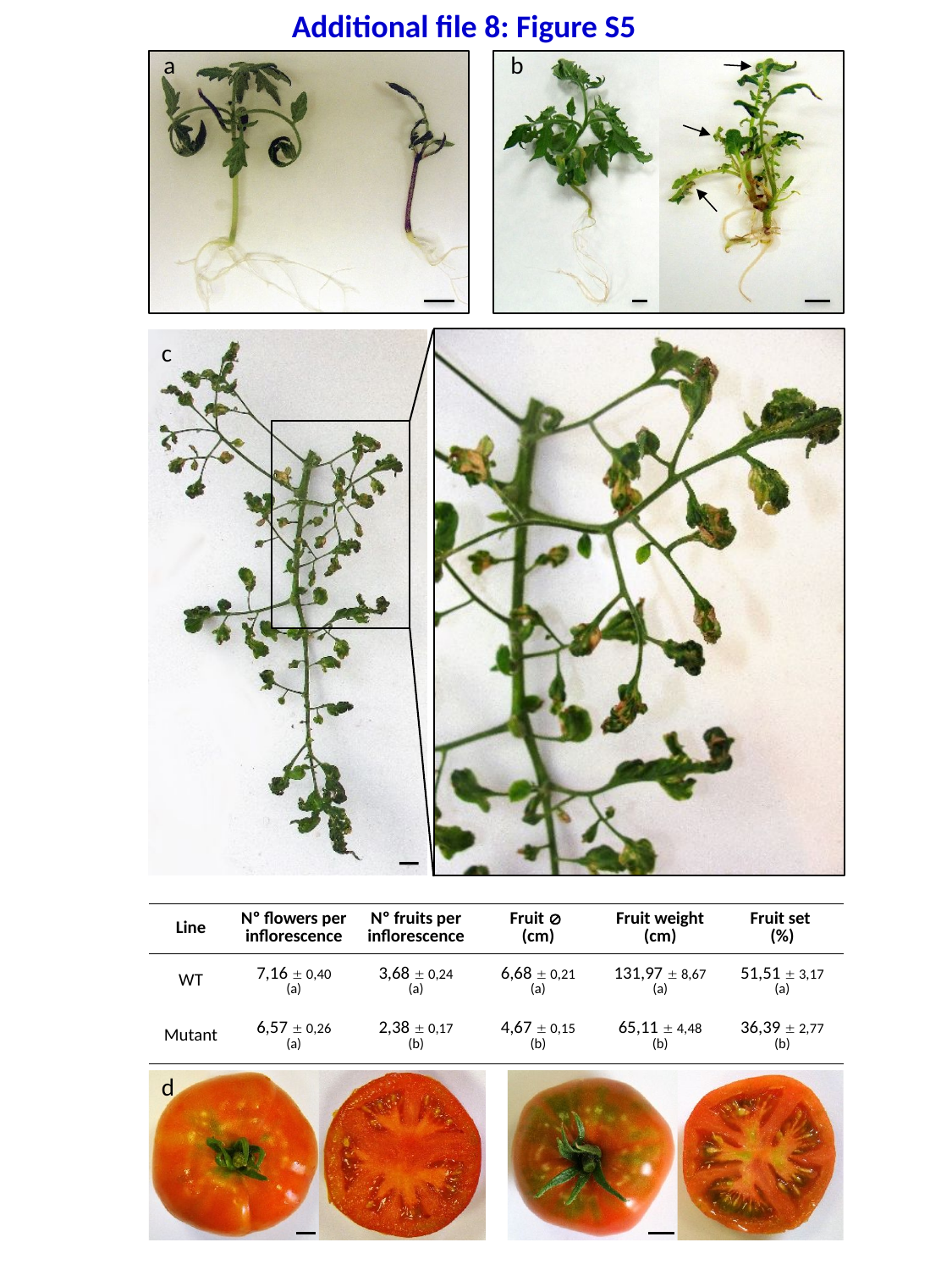

Additional file 8: Figure S5
a
b
c
| Line | Nº flowers per inflorescence | Nº fruits per inflorescence | Fruit  (cm) | Fruit weight (cm) | Fruit set (%) |
| --- | --- | --- | --- | --- | --- |
| WT | 7,16 ± 0,40 (a) | 3,68 ± 0,24 (a) | 6,68 ± 0,21 (a) | 131,97 ± 8,67 (a) | 51,51 ± 3,17 (a) |
| Mutant | 6,57 ± 0,26 (a) | 2,38 ± 0,17 (b) | 4,67 ± 0,15 (b) | 65,11 ± 4,48 (b) | 36,39 ± 2,77 (b) |
d
