# Supplementary material for: Phenotypic and genetic characterization of tomato mutants provides new insights into leaf development and its relationship to agronomic traits
Source: BMC Plant Biol. 2019 Apr 15;19:141. doi: 10.1186/s12870-019-1735-9 (PMC6466659; doi:10.1186/s12870-019-1735-9)
Supplement: Supplementary file 9 — Figure S6. Flowering time in plants of the mutant Altered in all reproductive traits (Art). a. Number of phytomers up to the first inflorescence (0–1) and between inflorescences (1–2, 2–3, …). b Length from base up to the first inflorescence (0–1) and between inflorescences (1–2, 2–3, …). (PPTX 63 kb) [file 12870_2019_1735_MOESM9_ESM.pptx]

## Slide 1
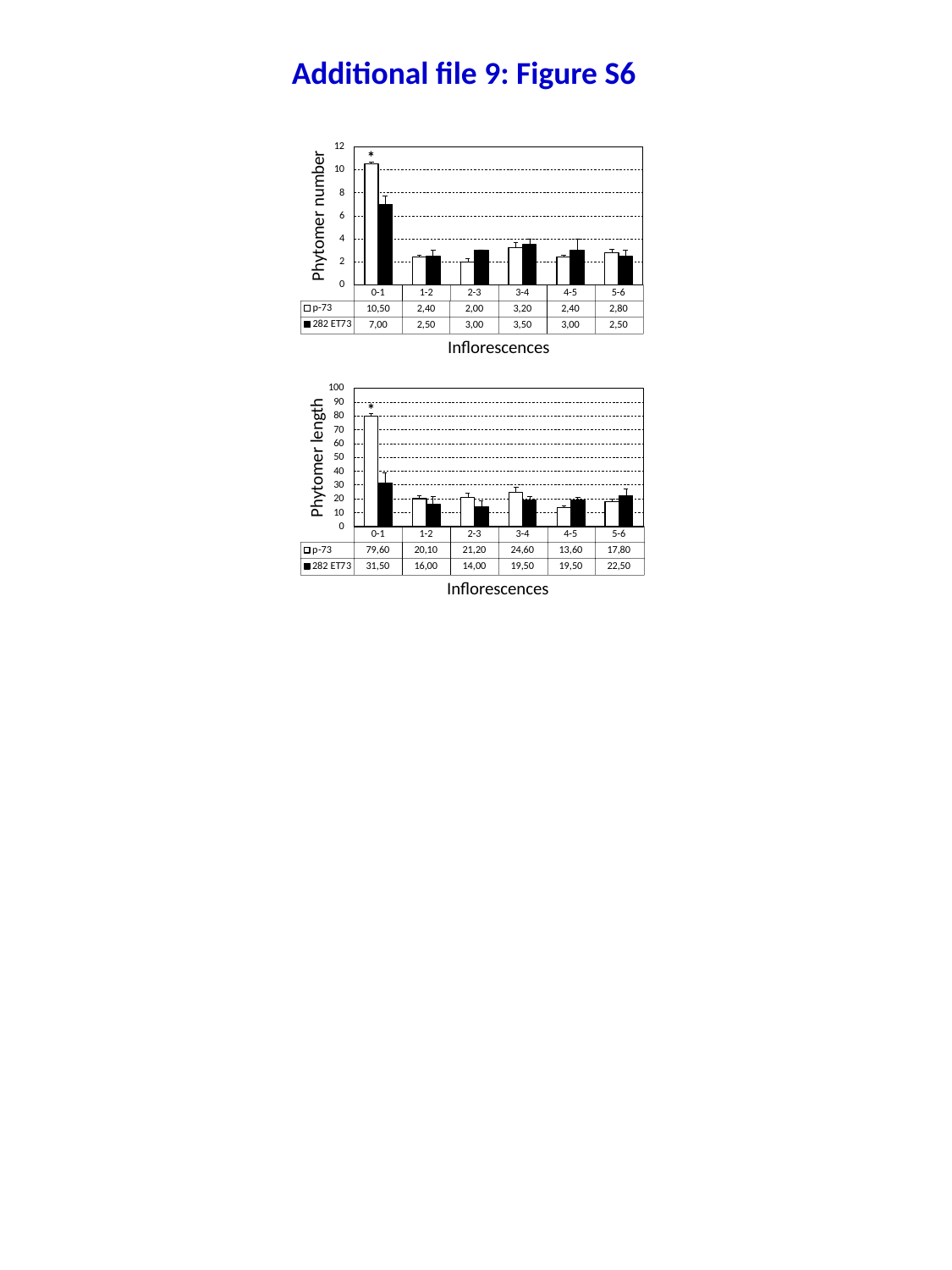

Additional file 9: Figure S6
*
Phytomer number
Inflorescences
*
Phytomer length
Inflorescences
